# Local generation of hydrogen for enhanced photothermal therapy

**DOI:** 10.1038/s41467-018-06630-2

**Published:** 2018-10-12

**Authors:** Penghe Zhao, Zhaokui Jin, Qian Chen, Tian Yang, Danyang Chen, Jin Meng, Xifeng Lu, Zhen Gu, Qianjun He

**Affiliations:** 1 Guangdong Provincial Key Laboratory of Biomedicalim Measurements and Ultrasound Imaging, National-Regional Key Technology Engineering Laboratory for Medical Ultrasound, School of Biomedical Engineering, Health Science Center, Shenzhen University, No. 1066 Xuyuan Road, Nanshan District, Shenzhen, 518055 Guangdong China; 2 Department of Bioengineering, University of California, Los Angeles, Los Angeles, CA 90095 USA; 3 California NanoSystems Institute, Jonsson Comprehensive Cancer Center, Center for Minimally Invasive Therapeutics, University of California, Los Angeles, Los Angeles, CA 90095 USA; 4 Joint Department of Biomedical Engineering, University of North Carolina at Chapel Hill and North Carolina State University, Raleigh, 27695 NC USA

## Abstract

By delivering the concept of clean hydrogen energy and green catalysis to the biomedical field, engineering of hydrogen-generating nanomaterials for treatment of major diseases holds great promise. Leveraging virtue of versatile abilities of Pd hydride nanomaterials in high/stable hydrogen storage, self-catalytic hydrogenation, near-infrared (NIR) light absorption and photothermal conversion, here we utilize the cubic PdH_0.2_ nanocrystals for tumour-targeted and photoacoustic imaging (PAI)-guided hydrogenothermal therapy of cancer. The synthesized PdH_0.2_ nanocrystals have exhibited high intratumoural accumulation capability, clear NIR-controlled hydrogen release behaviours, NIR-enhanced self-catalysis bio-reductivity, high NIR-photothermal effect and PAI performance. With these unique properties of PdH_0.2_ nanocrystals, synergetic hydrogenothermal therapy with limited systematic toxicity has been achieved by tumour-targeted delivery and PAI-guided NIR-controlled release of bio-reductive hydrogen as well as generation of heat. This hydrogenothermal approach has presented a cancer-selective strategy for synergistic cancer treatment.

## Abstract (web summary)

The tracking and targeted release of multi-agents to cooperatively treat cancer is a developing and evolving field. Here, the authors demonstrate the anticancer effects of cubic Pd hydride nanocrystals with photoacoustic imaging properties that can release hydrogen under an NIR trigger and have photothermal effects.

## Introduction

Hydrogen gas (H_2_), as a kind of endogenous gas, is not only an important energy source, but also exhibits significant physiological/pathological regulation functions^1^. As early as 1975, hydrogen gas has been found to be able to treat cancers^2^. In 2007, hydrogen was confirmed to be a therapeutic antioxidant that can selectively reduce cytotoxic oxygen radicals induced by ischaemia–reperfusion and inflammation^3,4^. In the past 10 years, hydrogen gas has been testified to practicably treat many diseases, including cancer, diabetes, stroke, atherosclerosis, Parkinson’s disease, Alzheimer’s disease, arthritis, dermatitis, colonitis, hepatitis, pancreatitis, myocardial infarction and tristimania^1–12^. The selective anti-oxidation profile of hydrogen gas is a commonly accepted mechanism for hydrogen therapy. As to cancer, both increase and decrease of rightly overexpressed reactive oxygen species (ROS) level in cancer cells could break the redox homoeostasis and bring redox stress, causing cancer cell damage and apoptosis^13,14^. The mechanism of hydrogen therapy for cancer could be related to the influence of hydrogen on intratumoural ROS level by virtue of its anti-oxidation. Besides good biomedical applicability, high bio-safety of hydrogen gas has also been widely confirmed^15–22^. Therefore, hydrogen therapy receives increasing attention with progressive clinical trials^23–29^, and is being accepted as a promising therapeutic method^30^.

Hydrogen gas has some special properties, including high aimless bio-diffusibility derived from small molecular size, nonpolarity and low solubility (1.6 ppm) at the physiological conditions. Therefore, the traditional administration routes, such as inhalation of hydrogen gas, oral intake of hydrogen-rich water and injection of hydrogen-rich saline, have undesirable performance for disease-targeted delivery and controlled release of hydrogen, frequently leading to limited therapeutic efficacy^7–12^. How to realize the effective storage, targeted delivery and controlled release of hydrogen is vitally important to enhance hydrogen therapeutic efficacy, but it still remains challenging at present^31,32^. Nanomaterial provides a promising platform to overcome this challenge^5,6,33–35^. Noticeably, Pd nanocrystal is an excellent hydrogen storage material and ideal catalyst for hydrogenation reaction^36–43^, potentially promoting hydrogen therapy outcomes. In this work, we design the Pd hydride (PdH_0.2_) nanocrystals as a multifunctional hydrogen carrier and Pd-similar self-catalyst to achieve the tumour-targeted delivery and controlled release of bio-reductive hydrogen.

The combination of gas therapy and traditional therapy, such as chemotherapy and radiotherapy, has proved to have synergetic therapy effects^44–52^. Pd nanomaterials with varied morphologies have been demonstrated as excellent photothermal therapy (PTT) and photoacoustic imaging (PAI) agents with high near-infrared (NIR)-photothermal conversion efficiencies and good biocompatibility^53–59^. Herein, we design the hydrogenated Pd nanocrystals to realize the combined hydrogenothermal therapy. Unexpectedly, we discovered that the hydrogenothermal therapy not only has a synergetic anticancer effect, but also potentially protects normal cells from hyperthermia damage.

**Fig. 1:**
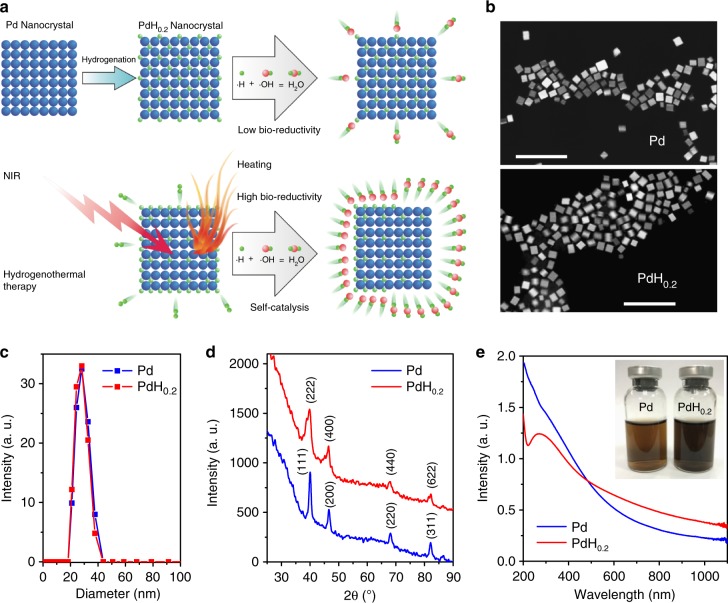
Synthesis and characterization of PdH_0.2_ nanocrystals. **a** Schematic illustration of the synthesis and NIR-controlled release of bio-reductive hydrogen and the generated heat from PdH_0.2_ nanocrystals for hydrogenothermal therapy. **b** SEM images of Pd and PdH_0.2_ nanocrystals. Scale bar, 100 nm. **c** DLS data of Pd and PdH_0.2_ nanocrystals. **d** XRD patterns of Pd and PdH_0.2_ nanocrystals. **e** UV−VIS−NIR spectra of Pd and PdH_0.2_ nanocrystals (60 μg mL^−1^)

In this work, we synthesize a 30 nm cubic Pd nanocrystal to achieve the passive tumour targeting by the enhanced permeability and retention (EPR) effect. Importantly, considerable hydrogen could be incorporated and stabilized within the lattice of Pd crystals to form Pd hydride (Fig. 1a). The hydrogenation of Pd nanocrystals results in the significant enhancement of NIR absorption, which enables the PAI-guided release of bio-reductive hydrogen as well as photothermal therapy. The tumour-targeted delivery and PAI-guided release of hydrogen and the generated heat demonstrate significantly enhanced anticancer efficacy.

## Results

### Synthesis and characterization of Pd and PdH_0.2_ nanocrystals

It has been well proved that Pd, especially in nanoscale, has the excellent capacity of hydrogen storage as well as thermal stability after incorporation of hydrogen into the lattice of Pd. Therefore, the nano-sized Pd crystals were synthesized and used as the hydrogen host to form PdH_0.2_. From Fig. 1b, c, it could be found that synthesized Pd nanocrystals exhibited cubic morphology, uniform particle size and good dispersion owing to the surface coating of poly(vinyl pyrrolidone) (PVP; Supplementary Fig. 1), and PdH_0.2_ completely inherited/unchanged these features in morphology and size. Such a small size and good dispersion are favourable for passive tumour targeting^60,61^. From X-ray diffraction (XRD) data in Fig. 1d, it could be found that both synthesized Pd and PdH_0.2_ were typical face-centred cubic crystals, and the unit cell parameters of Pd became a little bigger after hydrogenation, suggesting that hydrogen atoms had been incorporated into the lattice of Pd crystals forming Pd hydride^36^. Compared to Pd, the ultraviolet (UV)-zone absorption of PdH_0.2_ (Fig. 1e) became weaker but the VIS−NIR-zone absorption of PdH_0.2_ became stronger, enabling the PAI-guided release of hydrogen and enhanced photothermal therapy.

### Behaviour and mechanism of NIR-responsive hydrogen release

**Fig. 2:**
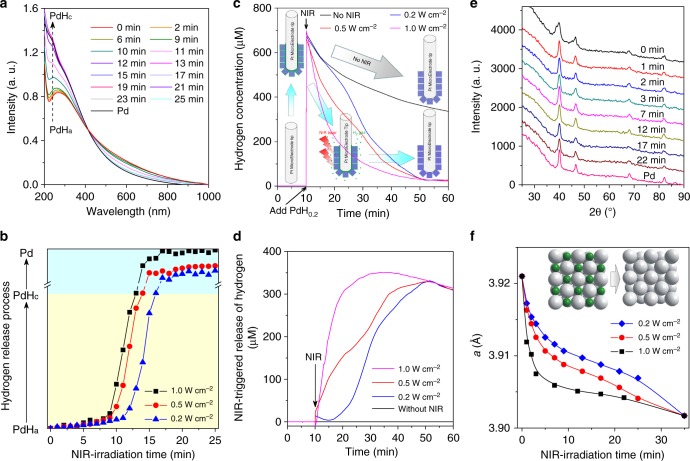
Process and mechanism of NIR-responsive hydrogen release from PdH_0.2_ nanocrystals. **a**−**f** Real-time monitoring of hydrogen release from PdH_0.2_ nanocrystals under the continuous irradiation of 808 nm NIR light by UV (**a**, **b**), Pt-microelectrode (**c**, **d**) and XRD (**e**, **f**) techniques. XRD tracing data at 1.0, 0.5 and 0.2 W cm^−2^ of NIR power densities were exhibited in **e**, Supplementary Fig. 3a and b, respectively

Furthermore, the process and mechanism of NIR-responsive hydrogen release from PdH_0.2_ were investigated by UV, XRD and Pt-microelectrode methods. According to the increase-to-decrease transformations of UV characteristic peak intensity (shown by up/down arrows in Supplementary Fig. 2 and Fig. 2a) under the continuous irradiation of 808 nm light, it was inferred that there could be a critical intermediate phase (represented by PdHc) during dehydrogenation of PdH_0.2_. According to the Beer–Lambert law, the hydrogen release percentage of PdH_0.2_ nanocrystals could be calculated (Supplementary Equations 7, 8), and the hydrogen release process could be quantitatively and intuitively demonstrated as shown in Fig. 2b. It was uncovered that the rate of the hydrogen release from PdH_0.2_ nanocrystals increased first and then decreased under the NIR irradiation, where there was a critical point (Fig. 2b). Such a dehydrogenation process is different from general drug release, but similar to the photochemical gas-releasing decomposition of CO prodrug previously reported by us, exhibiting a typical profile of photochemical reaction^62^. By respective irradiation at different power densities (0.2, 0.5, 1.0 W cm^−2^), it could be found that PdH_0.2_ was responsive to NIR light in a power density-dependent manner (Fig. 2b). Higher power densities of NIR light caused faster hydrogen release from PdH_0.2_. Therefore, it is feasible to control the rate and amount of hydrogen release by adjusting the power and duration of NIR irradiation.

Moreover, the Pt-microelectrode method was used to further quantitatively detect the hydrogen release from PdH_0.2_ nanocrystals in real time. Unexpectedly, large amounts of hydrogen could be detected by the Pt-microelectrode once PdH_0.2_ nanocrystals were injected into pure degassed water with the hydrogen microelectrode (50 μm in diameter), and then the detected hydrogen concentration decreased gradually in the absence of NIR irradiation (Fig. 2c). It was thought that the nano-sized Pt-microelectrode tip quickly adsorbed PdH_0.2_ nanocrystals and seized the active hydrogen atoms on the surface of PdH_0.2_ nanocrystals (as shown by green interface), and then consumed the adsorbed hydrogen gradually (as indicated by grey arrow). When the Pt-microelectrode was irradiated by NIR laser immediately after the addition of PdH_0.2_ nanocrystals, the detected hydrogen amount was obviously less compared to the case of no NIR irradiation (Fig. 2c), suggesting that hydrogen escaped from PdH_0.2_ nanocrystals under the NIR irradiation which led to less detectable interface active hydrogen (as shown by blue arrows) till the completion of hydrogen release (about 50 min of NIR irradiation). It could also be found that higher NIR power density resulted in less hydrogen detection (Fig. 2c), indicating that higher power density of NIR irradiation could cause faster hydrogen release from PdH_0.2_ nanocrystals in accordance with above-mentioned UV monitoring results. Taking the case of no NIR irradiation as background for subtraction, the NIR-triggered release of hydrogen from PdH_0.2_ nanocrystals could be represented to distinctly demonstrate the NIR-triggered hydrogen release behaviours of PdH_0.2_ nanocrystals, as shown in Fig. 2d. In addition, the XRD technique was also used to in-time track the change in the crystal structure of PdH_0.2_ under the irradiation of NIR light. From XRD data (Fig. 2e), it could be observed that XRD peaks of PdH_0.2_ were gradually shifted to Pd during the NIR irradiation. According to the Bragg equation, the unit cell parameter *a* was calculated (see Methods), as shown in Fig. 2f. It could be found that the unit cell parameter *a* of PdH_0.2_ gradually reduced to approach that of Pd during the continual NIR irradiation (Fig. 2f), indicating the NIR-responsive release of hydrogen from the lattice of PdH_0.2_. Furthermore, higher power of NIR irradiation caused sharper shrinkage of crystalline lattice, reflecting quicker release of hydrogen (Fig. 2f, Supplementary Fig. 3) in accordance with the above-mentioned UV/microelectrode tracing results.

### Bio-reductivity of NIR-responsive PdH_0.2_ nanocrystals

**Fig. 3:**
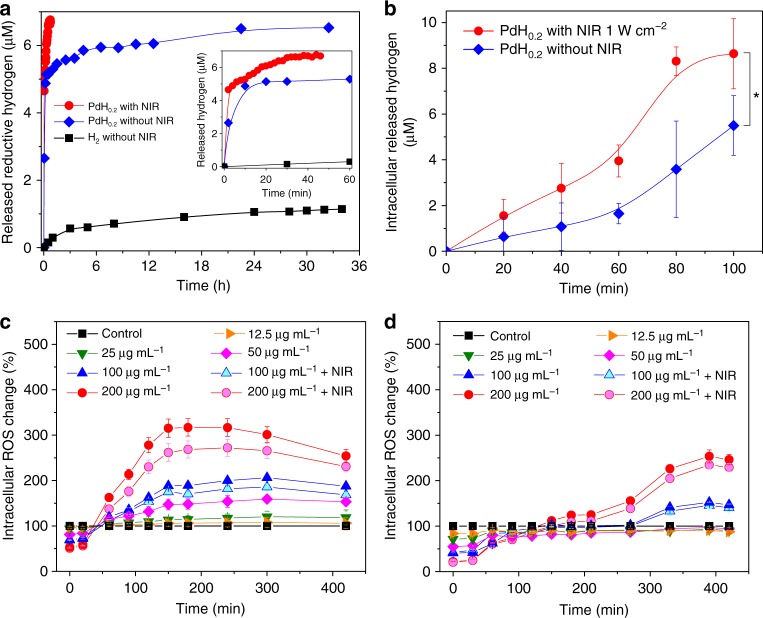
NIR-enhanced reduction capability of active hydrogen released from PdH_0.2_ nanocrystals. **a**, **b** Hydrogen release in stimulated body fluid (**a**) and in HeLa cells (**b**), which was detected using MB as an oxidative probe for active hydrogen. *P* values were calculated by two-tailed Student’s *t*-test (*P < 0.05). **c**, **d** The effect of PdH_0.2_ nanocrystals on intracellular ROS levels in cancer (HeLa cells, **c**) and normal (HEK-293T cells, **d**) cell models in the presence and absence of NIR irradiation (*n* = 6). In **a**, the treatment of MB with the H_2_-rich water was used as the blank control without PdH_0.2_ and NIR irradiation. Mean value and error bar are defined as mean and s.d., respectively

The bio-reductivity of released hydrogen from PdH_0.2_ and the effect of NIR irradiation were further investigated by methylene blue (MB), which was an oxidative probe for active hydrogen detection. Generally, MB needs to be combined with Pt nanoparticles as catalyst for hydrogen detection^63^. However, PdH_0.2_ nanocrystals could play the Pt-similar catalyst role (called self-catalyst) to assist the hydrogenation reaction (namely assist MB for hydrogen detection), which also means that the hydrogen released from PdH_0.2_ has the enhanced reductivity compared with free hydrogen gas (Fig. 3a). According to the hydrogen detection mechanism of the MB probe (Supplementary Fig. 4), the blue colour fading means the reduction of MB, reflecting the bio-reductivity of released hydrogen or hydrogen gas. Thus, the released reductive hydrogen could be quantified according to the change in the absorbance of the characteristic absorption peak of MB at 664 nm and the plotted standard curve by virtue of the Beer–Lambert law (Supplementary Fig. 5). The hydrogen-incorporation capacity of PdH_0.2_ was calculated to be about 0.2 mol mol^−1^, determining the actual molecular formula of synthesized Pd hydride to be PdH_0.2_. Moreover, hydrogen detection results indicated that the H_2_-rich water (self-made by blowing H_2_ gas into deionized water as shown in Supplementary Fig. 6) was discovered to be able to slowly reduce MB to a certain extent in the absence of any catalyst (Fig. 3a). By comparison with free hydrogen gas, PdH_0.2_ nanocrystals exhibited higher reductivity without NIR irradiation (Fig. 3a, Supplementary Fig. 6, 7) owing to the existence of hydrogen atoms on the crystal surface, confirming that PdH_0.2_ was remarkably enhanced under the NIR irradiation (Fig. 3a), owing to the NIR-responsive hydrogen release and the self-catalysis function of PdH_0.2_. In addition, the stability of PdH_0.2_ nanocrystals in phosphate-buffered saline (PBS) was further evaluated by in-time UV monitoring for 48 h in the absence of NIR irradiation. It was found that the UV spectrum was barely changed within 48 h (Supplementary Fig. 8), indicating a high stability of synthesized PdH_0.2_ under the present conditions in favour of NIR-controlled release of hydrogen. Such a relatively high stability of PdH_0.2_ nanocrystal was possibly attributed to its low chemical potential owing to its low hydrogen-incorporating capacity^64,65^. The bio-reductivity of PdH_0.2_ was further checked in a cell line model using the MB probe. As shown in Fig. 3b, PdH_0.2_ still exhibited the intracellular time-dependent bio-reductivity (Supplementary Fig. 9) similar to the case of the simulated body fluid. The NIR irradiation could similarly excitate PdH_0.2_ to release the bio-reductive hydrogen in cells and consequently enhanced the bio-reductivity of PdH_0.2_. Both results in simulated body fluid and cells confirmed that PdH_0.2_ nanocrystals had bio-reductivity, and it could be enhanced under the NIR irradiation.

The confirmed NIR-controlled bio-reductivity of PdH_0.2_ nanocrystals encouraged us to study their application for disease treatment. According to a previous study, Ohta and colleagues^3^ found that the administration of H_2_ could selectively reduce cytotoxic ROS, such as hydroxyl radical, which are generally overexpressed in tumour cells. The anti-oxidative ability of intracellular administrated H_2_ is one of the main mechanisms for hydrogen therapy of inflammation-related diseases. Therefore, the effect of PdH_0.2_ nanocrystals on intracellular ROS levels in cancer and normal cell models was investigated in order to evaluate its potential for cancer therapy. A ROS assay kit based on the dichlorodihydrofluorescein diacetate (DCFH-DA) probe was used to measure the change of intracellular ROS levels after treatment with PdH_0.2_ nanocrystals without/with the NIR irradiation. From Fig. 3c, d, it could be discovered that the administration of PdH_0.2_ nanocrystals immediately caused remarkable decrease of the intracellular ROS level in both cancer and normal cell models in a concentration-dependent way, further confirming the intracellular bio-reductivity of PdH_0.2_. Subsequently, the intracellular ROS levels in two cell models gradually increased (about 40 min and 120 min for cancer and normal cells, respectively), then decreased (150−300 min and 400 min for cancer and normal cells, respectively). Cancer cells were more sensitive to PdH_0.2_ nanocrystals than normal cells, possibly owing to the higher initial ROS level in cancer cells. The first increase of intracellular ROS level could be attributed to the stress response of cells to PdH_0.2_ nanocrystals, while the consequent decrease of intracellular ROS level should be due to the gradual release of bio-reductive hydrogen from PdH_0.2_ nanocrystals as the NIR irradiation could accelerate this process (Fig. 3c, d). It is worth noting that there was not only simple antagonistic effect between ROS and bio-reductive hydrogen from PdH_0.2_, but there were also differences in the ROS response to hydrogen and the redox homoeostasis of different cells. Remarkably higher redox stress was suppressed on cancer cells compared to normal cells. The reduction effect of PdH_0.2_ nanocrystals induced the intracellular ROS level to quickly decline, subsequently causing the rebound of ROS due to the redox homoeostasis capability of cells. Owing to the relatively higher ROS level in cancer cells, the initial ROS loss in cancer cells was higher and the subsequent ROS rebound was also intenser/higher than that in normal cells. The highly overexpressed ROS in cancer cells was hardly eliminated to the normal level, leading to the oxidative stress remarkably (Fig. 3c). However, the rebound of ROS in normal cells was much slower and slighter so that bio-reductive PdH_0.2_ nanocrystals could easily scavenge the weaker oxidative stress and pull ROS back to the normal level (Fig. 3d). The thermal generation from PdH_0.2_/Pd nanocrystals under the NIR irradiation would cause inflammation and induce cell apoptosis^66–70^. As to cancer cells, both hydrogen and heat increased the ROS level and therefore the hydrogenothermal combination was expected to induce the synergetic enhancement for inhibiting cancer cells. However, as to normal cells, the residual bio-reductive capacity of PdH_0.2_ nanocrystals could persistently scavenge the ROS generated from heat after elimination of the weak oxidative stress from the redox homoeostasis, in favour of protecting normal cells from the heating damage. Based on the selective enhancement of ROS in cancer cells and the effective scavenging of upregulated ROS in cancer cells, PdH_0.2_ nanocrystals could induce the apoptosis of cancer cells and reduce heating damage toward normal cells.

### NIR-photothermal effect and PAI performance

**Fig. 4:**
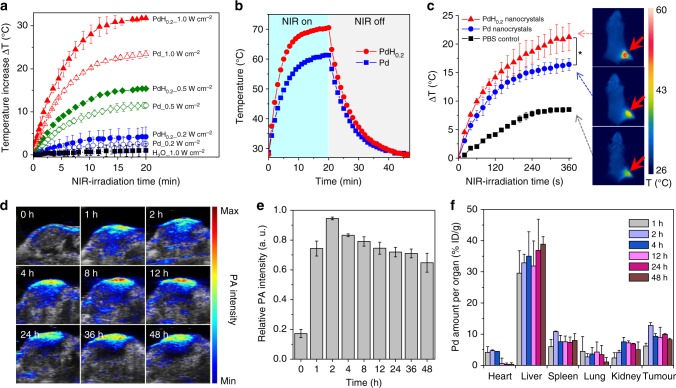
NIR-photothermal and photoacoustic effects of PdH_0.2_ nanocrystals. **a**  Photothermal effect of PdH_0.2_ and Pd nanocrystals in water (*n* = 3). **b** Photothermal efficiency evaluation of Pd and PdH_0.2_ solutions (60 μg mL^−1^) under irradiation of 808 nm laser at the power density of 1 W cm^−2^ which was turned off after irradiation for 20 min. **c** Photothermal imaging of tumour on 4T1 tumour-bearing mice (*n* = 3). **d** PAI images of 4T1 tumour treated with PdH_0.2_ nanocrystals (*n* = 3). **e**, **f** Quantitative analysis of intratumoural accumulation and retention of PdH_0.2_ nanocrystals from PAI (**e**) and ICP data (**f**). The aqueous solutions of PdH_0.2_ and Pd nanocrystals (40 μg mL^−1^) were irradiated by the 808 nm laser with different power densities (0.2, 0.5 and 1.0 W cm^−2^), and water without nanocrystals was used as the control to be irradiated by the 808 nm laser with the highest power density of 1.0 W cm^−2^. ICP data in (**f**) were collected at the different time points (1, 2, 4, 12, 24 and 48 h) post injection (*n* = 3). Mean value and error bar are defined as mean and s.d., respectively. *P* values were calculated by two-tailed Student’s *t*-test (*P < 0.05)

Besides hydrogen therapy, PdH_0.2_ nanocrystals could also be developed for combined hydrogenothermal therapy owning to their NIR absorption property (Fig. 1e). First, the NIR-photothermal effect of PdH_0.2_ nanocrystals was investigated under the 808 nm laser irradiation. From Fig. 4a, it could be found that the aqueous solution of PdH_0.2_ nanocrystals (40 μg mL^−1^) exhibited a clear NIR-photothermal effect, which was positively related to the power density and duration of NIR irradiation. Typically, the solution temperature increased by 13 °C after NIR irradiation at the power density of 0.5 W cm^−2^ for 10 min, which was sufficient to kill cancer cells. By comparison with Pd nanocrystals, PdH_0.2_ nanocrystals exhibited relatively stronger NIR-photothermal effects, owing to the more intensive NIR absorption (Fig. 1e). Furthermore, the photothermal conversion efficiencies (η) of PdH_0.2_ and Pd nanocrystals were measured (Fig. 4b) to be as high as 62.9% and 64.8%, respectively. Moreover, the NIR-photothermal effect of PdH_0.2_ nanocrystals in the 4T1 tumour model was checked. At 1 h after tail vein injection of PdH_0.2_ nanocrystals (2 mg mL^−1^, 100 μL), the mice were anesthetized, and then the tumours were irradiated by NIR light at the power density of 1.0 W cm^−2^ for 6 min (pointed by red arrows in Fig. 4c). The change of temperature at the tumour site was monitored using a thermal imaging camera. As shown in Fig. 4c, it was observed that the PBS control group showed about 8.5 °C of temperature increase at the tumour site, while the PdH_0.2_ and Pd groups exhibited about 21 °C and 16.5 °C of temperature increase, suggesting that PdH_0.2_ and Pd nanocrystals could effectively accumulate in the tumour via a passive targeting way and had enough high NIR-photothermal effect in vivo for thermal therapy of cancer.

Furthermore, PAI technique was applied to confirm the intratumoural accumulation of PdH_0.2_ nanocrystals and its PAI performance for therapy guidance. PdH_0.2_ nanocrystals were firstly confirmed to have excellent PAI performance in the PBS (Supplementary Fig. 10). Moreover, as shown in Fig. 4d−f, compared with pre-injection (0 h), the intratumoural PAI signal quickly increased at 1 h post vein injection of PdH_0.2_ nanocrystals and reached the maximum at 2 h, and then maintained at a relatively high level for 48 h. It is indicated that PdH_0.2_ nanocrystals could effectively accumulate and stably resorted in the tumour in accordance with the above-mentioned NIR-photothermal imaging results, and could also have excellent PAI performance in favour of tumour localization and therapy guidance. Moreover, the biodistribution and intratumoural accumulation of PdH_0.2_ nanocrystals 48 h after vein injection were further investigated by the inductively coupled plasma (ICP) measurement. From Fig. 4f, it could be found that the intratumoural accumulation of PdH_0.2_ nanocrystals at 2 h post injection is quite significant besides that associate with liver and spleen in support of passive tumour targeting by EPR effect. Nevertheless, no visible tissue toxicity to liver and spleen could be observed after treatment for 3 weeks (Supplementary Fig. 11).

### In vitro hydrogenothermal therapy efficacies and mechanisms

**Fig. 5:**
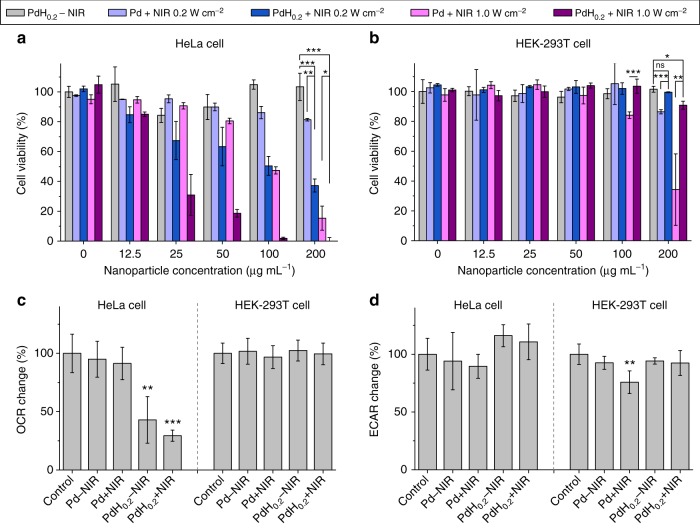
In vitro and In vivo therapy efficacies of PdH_0.2_ nanocrystals. **a**, **b** In vitro outcomes of combined hydrogenothermal therapy to HeLa cancer cells (**a**) and HEK-293T normal cells (**b**) (*n* = 8). **c**, **d** The mitochondrial (oxidative phosphorylation, **c**) and non-mitochondrial (glycolysis, **d**) metabolism behaviours of HeLa cells and HEK-293T cells (*n* = 4). Mean value and error bar are defined as mean and s.d., respectively. *P* values were calculated by two-tailed Student’s *t*-test (***P < 0.005, **P < 0.01, *P < 0.05), and ns represented no significance

Based on the above-confirmed bio-reductivity of NIR-responsively released hydrogen and NIR-photothermal effects of PdH_0.2_ nanocrystals, combined hydrogenothermal therapy of cancer was worthy of expectation and was further investigated in vitro and in vivo. Firstly, the cytotoxicities of PdH_0.2_ and Pd nanocrystals against cancer (4T1, B16-F10, HeLa cells) and normal (HEK-293T cells) cells were investigated and compared in vitro. From Supplementary Fig. 12a, e, i, m, PdH_0.2_ nanocrystals without the NIR irradiation generally exhibited the remarkable concentration- and time-dependent cytotoxicity against various cancer cells (Supplementary Fig. 12a, e, i), but insignificant cytotoxicity to normal cells in the investigated concentration range (0−200 μg mL^−1^) in spite of incubation time (Supplementary Fig. 12 m, Fig. 5a, b). It is suggested that PdH_0.2_ could selectively kill cancer cells rather than normal cells only by hydrogen therapy, possibly owing to the difference in the effects of the bio-reductive hydrogen from PdH_0.2_ nanocrystals on the intracellular ROS levels in cancer and normal cells as mentioned above (Fig. 3). In order to investigate thermal therapy effect, we applied Pd nanocrystals plus NIR irradiation as a control. As shown in Supplementary Fig. 12b, f, j, n, thermal therapy by Pd nanocrystals plus NIR irradiation had no selectivity to cell types, which killed both cancer and normal cells in a NIR power density-dependent and concentration-dependent way. However, the combined hydrogenothermal therapy by PdH_0.2_ nanocrystals plus NIR irradiation still exhibited remarkably an anticancer selectivity (Supplementary Fig. 12c, g, k, o). By comparison, the combined hydrogenothermal therapy by PdH_0.2_ nanocrystals plus NIR irradiation remarkably enhanced the anticancer efficacies of hydrogen therapy or/and thermal therapy to achieve a synergetic anticancer effect (Supplementary Fig. 12d, h, l), but had no remarkable cytotoxicity to HEK-293T normal cells (Supplementary Fig. 12p). Such cancer-selective, anticancer synergetic and normal-protective effects of hydrogenothermal therapy by PdH_0.2_ nanocrystals could be valuable for high-efficacy and low-toxicity cancer treatment.

In order to further clarify the mechanism of hydrogenothermal therapy, we investigated cell energy metabolism behaviours in addition to the alteration of ROS level in cancer and normal cells (Fig. 5c, d). An illustration (Supplementary Fig. 14) was also drawn to elucidate the mechanism. It could be found that PdH_0.2_ nanocrystals without NIR irradiation did damage to the mitochondrial metabolism function of cancer cells (ATP↓, Fig. 5c, Supplementary Fig. 13) which was reflected by oxygen consumption rate (OCR) so that cancer cells had to resort to more non-mitochondrial metabolism which was reflected by extracellular acidification rate (ECAR), and had depressed maximum respiration and increased spare respiratory capacity (Supplementary Fig. 13a, b). The damage to the mitochondria of cancer cells was possibly attributed to the increase of intracellular ROS level (Fig. 3c). On the other way, the ROS level in normal cells could be easily normalized (Fig. 3d) so that PdH_0.2_ nanocrystals had no obvious damage to mitochondrial function (ATP↑, Fig. 5d) and insignificant influence on energy metabolism of normal cells (OCR/ECAR→). The treatment with Pd nanocrystals under NIR irradiation was used to represent only thermal effect. It could be found that only thermal therapy always caused the overall inhibition to cell energy metabolism in both cancer and normal cells (Fig. 5c, d). By combination of hydrogenothermal therapy using PdH_0.2_ nanocrystals, the overall energy metabolism of cancer cells was blocked synergistically by hydrogen and heat (Fig. 5c, d), which resulted in enhanced cancer cell apoptosis (Fig. 5a). However, the strong ROS-scavenging effect of hydrogen maintained the balance of ROS level in normal cells and recovered their energy metabolism capability from heating damage (Fig. 5b−d, Supplementary Fig. 13c, d). Therefore, hydrogenothermal combination could selectively protect normal cells and enhance anticancer effect (Supplementary Fig. 14).

### In vivo hydrogenothermal therapeutic efficacies

**Fig. 6:**
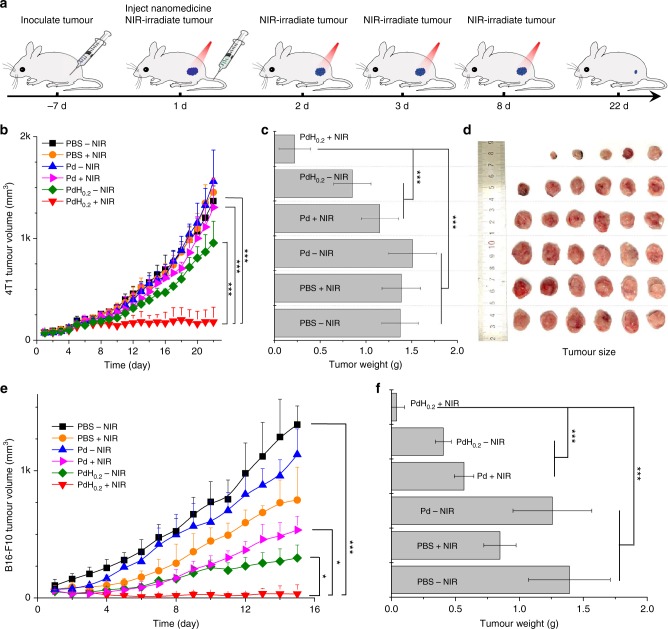
In vitro and in vivo therapy efficacies of PdH_0.2_ nanocrystals. **a** Tumour-bearing mice therapy approach. **b** 4T1 tumour volume change during treatment. **c** 4T1 tumour weight comparison after 22-day treatment. **d** Digital images of corresponding 4T1 tumours (*n* = 6). **e** B16-F10 tumour volume change during treatment. **f** B16-F10 tumour weight comparison after 15-day treatment (*n* = 6). Mean value and error bar are defined as mean and s.d., respectively. *P* values were calculated by two-tailed Student’s *t*-test (***P < 0.005, *P < 0.05) by comparing other groups with the PdH_0.2_ + NIR group

To further investigate in vivo hydrogenothermal therapeutic efficacy, we first injected healthy mice (about 20 g) with different concentrations of 100 μL PdH_0.2_ nanocrystals (2−100 mg mL^−1^, equal to 10−500 mg kg^−1^) to determine the safe dose. No mouse died after intravenous injection of PdH_0.2_ in the present concentration range (10−500 mg kg^−1^) for 8 days. The blood test results also indicated that PdH_0.2_ had not affected the liver and kidney functions of treated mice obviously even at the highest injection dose of 500 mg kg^−1^ (Supplementary Fig. 15). However, PdH_0.2_ could not bring visible abnormality of several blood indicators such as red blood cells (RBC), white blood cells (WBC), haemoglobin (HGB), means corpuscular haemoglobin concentration (MCHC), lymphocytes percentage (LYM), means corpuscular volume (MCV), haematocrit (HCT), and red blood cell volume distribution width (RDW-SD) until the dose of PdH_0.2_ went up to 500 mg kg^−1^ (Supplementary Fig. 16). It could be concluded that the safe dose of PdH_0.2_ nanocrystals was not lower than 250 mg kg^−1^. Therefore, the present injection dose of 10 mg kg^−1^ for cancer therapy should be safe enough. The 4T1 tumour model was established by subcutaneous injection of murine 4T1 breast cancer cells into the hind limb space of BALB/c (4 weeks old) mice. Mice were randomly divided into six groups: the PBS group (blank control), the PBS + NIR group (NIR control), the Pd−NIR group (Pd control), the Pd + NIR group (thermal therapy group), the PdH_0.2_−NIR group (hydrogen therapy group) and the PdH_0.2_ + NIR group (hydrogenothermal therapy group). As to NIR irradiation groups, the tumour site of mouse was exposed to NIR light (0.5 or 1 W cm^−2^) at fixed time points (days 1, 2, 3, 8) after 1 h post injection of PBS, Pd or PdH_0.2_ nanocrystals (100 μL, 10 mg kg^−1^), as illustrated in Fig. 6a. From Fig. 6b (0.5 W cm^−2^) and Supplementary Fig. 17 (1 W cm^−2^), neither only NIR irradiation nor only Pd nanocrystals without NIR irradiation affected the tumour growth, and both the hydrogen therapy group (PdH_0.2_−NIR) and the thermal therapy group (Pd + NIR) caused slight inhibition to the tumour growth. The most noticeable was that the combined hydrogenothermal therapy group (PdH_0.2_ + NIR) generated considerably remarkable tumour inhibition effect during the 22-day observation (Fig. 6b). It was also worth noticing that even though no more NIR irradiation was provided for thermal therapy after 8 day post injection, the tumours were still stably inhibited at a very small volume in the later period (days 8−22), indicating that later hydrogen therapy could maintain a good tumour inhibition efficacy owing to the super-sustained hydrogen release profile of PdH_0.2_ nanocrystals (Fig. 3a). Compared with only hydrogen therapy and only thermal therapy, combined hydrogenothermal therapy exhibited a synergetic anticancer effect (Fig. 6b) in accordance with the above-mentioned in vitro cytotoxicity results (Fig. 5a, b). After 22 days post injection, the tumours and main organs of mice were extracted to further confirm the hydrogenothermal therapy outcome and tissue compatibility of PdH_0.2_ nanocrystals (Fig. 6c, d). It was found that the differences in the mouse body weight among various treatment groups were insignificant (Supplementary Fig. 18 and 19). In addition, the histopathological evaluation by the haematoxylin and eosin (H&E) staining method (Supplementary Fig. 11) indicated that all treatment groups did not do visible damage to main organs (heart, liver, spleen, lung and kidney), reflecting good tissue compatibility of PdH_0.2_ nanocrystals.

Next, we further investigated another tumour model (B16-F10 melanoma tumour) which was established by subcutaneously injecting B16-F10 melanoma cancer cells into the hind limb space of BALB/c nude mice (4 weeks old). Relatively lower laser power density of 0.5 W cm^−2^ was used to irradiate the B16-F10 tumours owing to higher self-photothermal effect of melanoma tumour compared to 4T1 tumour. Even so, more obvious photothermal therapy outcomes were achieved (PBS + NIR and Pd + NIR groups, Fig. 6e, f). Melanoma tumour seems to be more sensitive to individual hydrogen therapy compared to 4T1 tumour (PdH_0.2_−NIR groups, Fig. 6e, f). Compared with only hydrogen therapy and only thermal therapy, combined hydrogenothermal therapy also exhibited a synergetic anticancer effect to B16-F10 melanoma tumour-bearing mice (Fig. 6e) in accordance with the above-mentioned outcome of 4T1 breast tumour-bearing mouse therapy (Fig. 6b). The tumour extraction results after 15-day treatment further conformed the remarkable hydrogenothermal therapy outcome of PdH_0.2_ nanocrystals (Fig. 6f).

## Discussion

In this work, we developed PdH_0.2_ nanocrystals for the tumour-targeted delivery and controlled release of bio-reductive hydrogen for hydrogenothermal therapy of cancer. PdH_0.2_ nanocrystals displayed a broad UV−to−NIR absorption spectrum with a stronger NIR absorption compared with the same size of Pd nanocrystals, and exhibited the enhanced NIR-photothermal effect, enabling the PAI-guided NIR-controlled release of hydrogen and the generated heat. PdH_0.2_ nanocrystals exhibited the selective and synergetic effects for hydrogenothermal therapy in vitro and in vivo. Of note, by combined hydrogenothermal therapy, the side effect of pure thermal therapy to normal cells/tissues could be reduced while its positive effect to cancer cells could be enhanced by the controlled administration of bio-reductive hydrogen. This broad-spectrum and safe therapy strategy could be highly desired for clinical applications. Such homoeostatic regulation function of bio-reductive hydrogen is similar to several typical therapeutic gases such as NO and CO, which could be explained from the standpoint of cell energy metabolism^71^, while hydrogen gas is highly bio-safe without obvious poisoning effect^15–22^.

## Methods

### Chemicals

Sodium tetrachloropalladate (Na_2_PdCl_4_), PVP, potassium bromide (KBr), *L*-ascorbic acid (AA), MB and sodium borohydride (NaBH_4_) were purchased from Sigma-Aldrich. The CCK-8 kit and the ROS assay kit were purchased from Beyotime Biotechnology Co., Ltd. All other agents used were of the highest commercial grade available.

### Synthesis of Pd nanocrystals

The Pd nanocrystals were synthesized by a facile redox route. Firstly, an aqueous solution (11 mL) of PVP (106.4 mg), AA (60 mg), KBr (301 mg) and Na_2_PdCl_4_ (56.31 mg) was heated at 80 °C under magnetic stirring for 3 h, and then cooled to room temperature. Then, the Pd nanocrystals were collected and purified using Amicon hyperfiltration tubes (molecular weight cut-off 100 kDa, Millipore) by 30 min of centrifugation at 1492 × *g* and three-time washing. Finally, the obtained Pd nanocrystals were dispersed in 10 mL of deionized water and stored in the dark for following use.

### Synthesis of PdH_0.2_ nanocrystals

The 2 mL Pd nanocrystals were injected into a vial of 20 mL and sealedt with a rubber stopper. Then, 100 mg NaBH_4_ were taken in another vial and sealed with a rubber stopper. Two vials were connected with a capillary. A needle was inserted connected with 1 mL syringe into the first vial for the connection to the atmosphere. Then, pH = 5 sulphuric acid solution was injected into the second vial containing NaBH_4_ using a syringe of 2 mL. NaBH_4_ in the presence of acidic solution can produce hydrogen gas, so that hydrogen gas could slowly drum into the Pd nanocrystal solution. After 15 min, the needle and capillaries were removed, the vial of PdH_0.2_ nanocrystals was sealed and stored in the dark for the further use.

### Characterization of Pd and PdH_0.2_ nanocrystals

The morphology and size of PdH_0.2_ and Pd nanocrystals were measured by scanning electron microscopy (SEM). The hydrodynamic size was measured on a Malvern Zetasizer Nano ZS90 equipped with a solid-state He-Ne laser (λ = 633 nm). The phase structures of PdH_0.2_ and Pd nanocrystals were characterized by powder XRD using a M21X diffractometer (Cu Kα, λ = 1.54056 Å) operated at 40 kV and 200 mA. The experimental diffraction patterns were collected with a scanning range of 5°−80° at room temperature. The composition of PdH_0.2_ nanocrystals was tested by an attenuated total reflectance Fourier transform infrared spectroscopy (ATR-FTIR). Concentrated sample solutions were dropped on the universal diamond ATR sampling accessory and slowly dried by blowing with nitrogen gas. FTIR spectra were then collected on a Thermo-Nicolet Nexus 670 ATR-IR spectrometer. The UV absorption spectra of the solution (200–1100 nm) were recorded at room temperature on a Genesys 10 s UV–visible (Vis) spectrophotometer (Thermo Sci.).

### Measurement of hydrogen release behaviour of the PdH_0.2_ nanocrystals by in-time UV and XRD monitorings

As to the UV monitoring, 3 mL 0.04 mg mL^−1^ PdH_0.2_ nanocrystal solution was placed into the cuvette, the solution was irradiatedn using an 808 nm NIR laser (1 W cm^−2^), and we immediately measured the UV spectra of the solution once every minute. From the change of the UV spectra (the band intensity below 410 nm increased firstly (Supplementary Fig. 2a) and then decreased (Supplementary Fig. 2b) with hydrogen release), we primarily decided that there could be a critical intermediate phase (PdHc). Therefore, the decomposition process of PdH_0.2_ was divided into two steps. According to the in-time detected UV data (Fig. 2a), the release percentage of hydrogen can be calculated (see Supplementary Discussion for calculation details), and plotted as a function of reaction time, as shown in Fig. 2b. In addition, the stability of PdH_0.2_ nanocrystals was evaluated by in-time UV monitoring for 48 h in the absence of NIR irradiation.

As to the XRD monitoring, PdH_0.2_ nanocrystals were uniformly coated on the sample plate by dropping and drying their ethanol solution. The sample plate was continuously irradiated by the 808 nm NIR laser (1 W cm^−2^). Meanwhile, a cascade of XRD patterns was collected (Fig. 2e). The interplanar spacing distance (*d*) was calculated according to the Bragg equation: 2*d*sinθ = *nλ* (*λ* = 0.154178 nm, *n* = 1, 2, 3…). Then, the unit cell parameter (*a*) could be obtained by the following equation: *a* = *d*(*h*^2^ + *k*^2^ + *l*^2^)^1/2^ (note that this equation applies to face-centred cubic crystals like our Pd crystals). The change in the unit cell parameter with time (Fig. 2f) was used to determine the crystal structure shrinking and the escape/release of hydrogen from the lattice of PdH_0.2_.

### Measurement of the release of reductive hydrogen from the PdH_0.2_ nanocrystals in PBS using the MB probe

Blue MB can be quickly reduced into colourless MBH_2_ by H_2_ under the catalysis of Pt nanocrystals, as shown in Supplementary Fig. 4. By virtue of this feature, the MB-Pt probe is frequently used for detection of H_2_ concentration according to the colour change during the hydrogenation of MB. In this work, we found that both Pd and PdH_0.2_ crystals have the similar catalytic hydrogenation effect to Pt nanocrystals, and therefore MB can be used as the Pt-free probe to in situ detect the hydrogen release from PdH_0.2_ nanocrystals. The reduction in the absorbance at 664 nm (the most characteristic peak of MB) is linearly related to the amount of H_2_. Therefore, the standard curve of the MB solution was measured using the UV spectrophotometer in order to quantify the reduction of MB and the release of H_2_, as shown in Supplementary Fig. 5. The detection measurement was executed according to a typical process as follows. Firstly, the MB probe solution (300 μg mL^−1^) was prepared. Then PdH_0.2_ nanocrystals (final 4 μg mL^−1^) were dispersed into the MB probe solution (final 3 mL) in a cuvette, which was monitored in real time by the UV measurement. It is noticeable that the concentration of MB should not exceed the detection range of UV, and should also be high enough to completely absorb all released hydrogen from PdH_0.2_ nanocrystals. Release experiments were divided into two groups, with NIR irradiation and without NIR irradiation. The group with the NIR irradiation was continuously irradiated till the balance (44 min) using the 808 nm NIR laser (1 W cm^−2^), and meanwhile was monitored by the UV spectrophotometer (Supplementary Fig. 6a). However, the other group without the NIR irradiation was monitored for about 33 h when the reaction was almost completed (Supplementary Fig. 6b).

### Cell culture

The mouse 4T1 breast cancer cells, B16-F10 melanoma cells, human HeLa cervical carcinoma cells and HEK-293T emborynic kidney cells were purchased from China Type Culture Collection (CTCC) obtained from the American Type Culture Collection (ATCC). Cells were routinely tested for mycoplasma contamination using MycoSET Mycoplasma real-time PCR detection Kit (Life Technologies, Foster City, CA, USA). They were cultured in Dulbecco's modified Eagle's medium (DMEM) with 10% (v:v) foetal bovine serum, 100 U mL^−1^ penicillin, and 100 μg mL^−1^ streptomycin in an incubator (Thermo Scientific) at 37 °C under the atmosphere of 5% CO_2_ and 90% relative humidity. To digest cells and subculture, 0.25% (w:v) trypsin was used.

### Measurement of intracellular hydrogen release and bio-reducibility of the PdH_0.2_ nanocrystals using the MB probe

Firstly, the other standard curve of the MB solution at 664 nm was measured in 96-well plate using a Bio-Tek microplate reader (Supplementary Fig. 7), which was used to quantify the intracellular reduction of MB and the intracellular release of H_2_, as the absorption intensity from microplate reader is different from that from UV spectrophotometer. HeLa cells at the density of 1 × 10^4^ cells/well were plated in a 96-well plate and cultivated with a DMEM culture medium containing 10% foetal bovine serum at 37 °C in a humidified and 5% CO_2_ incubator. After incubation for 24 h in 100 μL culture medium per well, the culture medium was replaced with fresh one containing the MB probe. After incubation for 1 h, the medium was replaced again with fresh one containing the PdH_0.2_ nanocrystals at the concentration of 100 μg mL^−1^. Then, one part of the 96-well plate was irradiated with the NIR laser of 1 W cm^−2^, and another part kept in the dark environment as the control. The next whole process was monitored in real time using the Bio-Tek multi-mode microplate reader (absorption wavelength: 664 nm) and using the microscopy (Supplementary Fig. 9).

### Measurement of the ROS level in cells

HeLa or HEK-293T cells at the density of 1 × 10^4^ cells were plated in each well of the 96-well plate and cultivated with the DMEM culture medium containing 10% foetal bovine serum at 37 °C in a humidified and 5% CO_2_ incubator. After incubation for 24 h in 100 μL culture medium per well, the culture medium was replaced with fresh ones containing the PdH_0.2_ nanocrystals at final concentrations of 12.5–200 μg mL^−1^. After incubation for 1 h, the wells at the concentration of 200 μg mL^−1^ and 100 μg mL^−1^ (as the experiment group) were irradiated with the NIR laser of 1 W cm^−2^. The rest was kept in the dark (as the control group). Then, 10 μL DCFH-DA was added into each well and monitored using the Bio-Tek multi-mode microplate reader (488 nm excitation wavelength, 525 nm emission wavelength). In addition, the wells without the PdH_0.2_ nanocrystals served as the blank control (background). Experiment groups extracted the background to obtain the normalized ROS values for indicating the change of intracellular ROS level.

### Cytotoxicity measurement of the PdH_0.2_ nanocrystals

The mouse 4T1 breast cancer cells, B16-F10 melanoma cells, human HeLa cervical carcinoma cells and HEK-293T emborynic kidney cells were purchased from CTCC obtained from the ATCC. Cells were routinely tested for mycoplasma contamination using MycoSET Mycoplasma real-time PCR detection Kit (Life Technologies, Foster City, CA, USA). The cells (1 × 10^4^ cells/well) were planted in the 96-well plate, and co-incubated with the PdH_0.2_ or Pd nanocrystals at final concentrations of 12.5–200 μg mL^−1^ with or without the NIR irradiation. The following treatment groups (*n* *=* 8) were used for comparison: two control groups, 3 h of incubation with PBS or Pd nanocrystals without the NIR irradiation; only PTT therapy group, 3 h of incubation with Pd nanocrystals with the 808 nm NIR irradiation at 1.0 or 0.2 W cm^−2^ for 10 min each well; only hydrogen therapy group, 3 h of incubation with PdH_0.2_ nanocrystals without the NIR irradiation; combined hydrogenothermal therapy group, 3 h of incubation with PdH_0.2_ nanocrystals with the 808 nm NIR irradiation at 1.0 or 0.2 W cm^−2^ for 10 min each well. After incubation for 24 h, the cellular viability was tested by the CCK-8 method. Mean value and error bar were defined as mean and s.d., respectively.

### Photothermal effects of Pd and PdH_0.2_ nanocrystals

The photothermal heating curves were obtained by monitoring the temperature change of sample solutions under the irradiation of 808 nm NIR laser at different power densities (0.2−1.0 W cm^−2^). The laser was provided by a fibre-coupled continuous semiconductor diode laser (KS-810F-8000, Kai Site Electronic Technology Co., Ltd.) and the temperature was recorded by a fixed-mounted thermal imaging camera (FLIR A300-series). The photothermal conversion efficiencies (*η*) of Pd and PdH_0.2_ nanocrystals were calculated using the method of Roper et al.^72^.

### In vivo photothermal imaging (PTI)

The 4T1 tumour-bearing mice model was established by injecting 1 × 10^7^ 4T1 cells into the hind limb of each female BALB/c mouse (~20 g, purchased from Guangdong Medical Laboratory Animal Center). After the mean volume of the tumours reached about 100 mm^3^, two experienced researchers randomly divided the mice into three groups (*n* = 3 per group). The tumour-bearing mice were injected with 100 μL of PBS (group 1) or 10 mg kg^−1^ Pd nanocrystals (group 2) or 10 mg kg^−1^ PdH_0.2_ nanocrystals through the vein of tail (group 3). After 2 h of injection, the mice were irradiated with the 808 nm laser at 1 W cm^−2^ for 6 min. During the course of irradiation, we utilized the infrared thermal imaging cameras (FLIR A300-series) to monitor the temperature change of the tumour sites. Group 1 was taken as the control. The Administrative Committee on Animal Research in Shenzhen University approved the protocols for all animal experiments.

### In vivo PAI

The 4T1 tumour-bearing mice model was established by injecting 1 × 10^7^ 4T1 cells into the hind limb of each female BALB/c mouse (~20 g, purchased from Guangdong Medical Laboratory Animal Center). All PAI experiments were performed on an optoacoustic imaging system (Vevo 2100 LAZR system, VisualSonic Inc.). For in vivo PAI study, the 4T1 tumour-bearing BALB/c mice were anesthetized by inhalation of 1% isoflurane, and then injected with the PdH_0.2_ nanocrystals (100 μL, 2 mg mL^−1^) via the tail vein. In vivo PA images were collected before injection and post injection at different time points (1, 2, 4, 8, 12, 24, 36 and 48 h). The average PA signal in the tumour tissue was extracted using the Vevo Lab software.

### Biodistribution analysis

The 4T1 tumour-bearing mice model was used to measure the biodistribution of PdH_0.2_ nanocrystals. After the tumour volume reached about 100 mm^3^, the 4T1 tumour-bearing mice were injected with 100 μL PBS solution (10 mg kg^−1^) of PdH_0.2_ nanocrystals. Eighteen mice were selected and each three mice were dissected separately at 1, 2, 4, 12, 24 and 48 h after drug injection. Besides, the excretion of mice were collected and weighed in the group of 48 h. The weights of the heart, liver, spleen, lung, kidney and tumour of each mouse were measured. Then, these organs were digested with aqua regia, heated to dryness and added to a certain volume with deionized water. The quantitative analysis of Pd element was determined by inductively coupled plasma-atomic emission spectrometry (ICP-AES, Agilent Technologies, USA).

### In vivo tumour therapy

All healthy female BALB/c mice (4 weeks old) were purchased from Guangdong Medical Laboratory Animal Center and all the in vivo experiments followed the protocols approved by the Animal Care and Use Committee of the Shenzhen University. For 4T1 tumour therapy, when the tumour size reached approximately 60−100 mm^3^ (designed as day 0), the treatment was performed. Two experienced researchers randomly divided the mice into six groups (*n* = 6 per group for 4T1 tumour), which were in situ injected with 100 μL PBS without and with 808 nm laser irradiation (as two blank controls, group 1 and group 2), 10 mg kg^−1^ Pd nanocrystals (as thermal therapy control, group 3), 10 mg kg^−1^ PdH_0.2_ nanocrystals (as hydrogen therapy group, group 4), 10 mg kg^−1^ Pd with 808 nm laser irradiation (as thermal therapy group, group 5), 10 mg kg^−1^ PdH_0.2_ with 808 nm laser irradiation (as hydrogenothermal therapy group, group 6) on days 1, 2, 3 and 8. Groups 2, 5 and 6 were treated with 808 nm laser at 0.5 or 1.0 W cm^−2^ for 5 min at tumour sites after 1 h of injection. The body weight and tumour size of each mouse were recorded every other day. The mice were humanely killed after 22 days of treatment and all the tumours were collected.

For B16-F10 tumour therapy, the B16-F10 tumour-bearing mice model was established by injecting 1 × 10^7^ B16-F10 cells into the hind limb of each female BALB/c nude mouse (~15 g, purchased from Guangdong Medical Laboratory Animal Center). When the tumour size reached approximately 60 mm^3^ (designed as day 0), the treatment was performed. Two experienced researchers randomly divided the mice into six groups (*n* = 6 per group), which were in situ injected with 100 μL PBS without and with 808 nm laser irradiation (as two blank controls, group 1 and group 2), 10 mg kg^−1^ Pd (as thermal therapy control, group 3), 10 mg kg^−1^ PdH_0.2_ (as hydrogen therapy group, group 4), 10 mg kg^−1^ Pd with 808 nm laser irradiation (as thermal therapy group, group 5), 10 mg kg^−1^ PdH_0.2_ with 808 nm laser irradiation (as hydrogenothermal therapy group, group 6) on days 1, 2, 3 and 8. Groups 2, 5 and 6 were treated with 808 nm laser at 0.5 W cm^−2^ for 5 min at tumour sites after 1 h of injection. The body weight and tumour size of each mouse were recorded every other day. The nude mice were humanely killed after 15 days of treatment and all the tumours were collected.

### H&E-stained histology

For the H&E-stained histological evaluation, main organs (heart, liver, spleeny, lung and kidney) were harvested after 3 weeks of treatment, fixed in a 4% polyoxymethylene solution and then embedded in paraffin for the H&E staining.

### The liver/kidney function and hemotoxicity analyses

Two experienced researchers randomly divided the BALB/c mice into 6 groups (*n* = 4 per group), which were intravenously injected with 100 μL PBS (as control, group 1), 100 μL 10 mg kg^−1^ PdH_0.2_ nanocrystals (group 2), 100 μL 20 mg kg^−1^ PdH_0.2_ nanocrystals (group 3), 100 μL 75 mg kg^−1^ PdH_0.2_ nanocrystals (group 4), 100 μL 250 mg kg^−1^ PdH_0.2_ nanocrystals (group 5) and 100 μL 500 mg kg^−1^ PdH_0.2_ nanocrystals (group 6). After 8 days, the blood of each mouse was taken and detected by biochemical analyzer (iMagic-M7) and blood cell analyzer (BC-31 s, Mindray).

### Cellular energy metabolism

HeLa or HEK-293T cells (3 × 10^4^ cells/well) were planted in the specific 24-well plate. After incubation for 4 h, another 150 μL culture medium containing the PdH_0.2_ or Pd nanocrystals at the final concentration of 100 μg mL^−1^ was added. To study the energy metabolism, we divided into 5 groups as follows: (1) two control groups, 24 h of incubation with culture medium or Pd nanocrystals; (2) only PTT therapy group, 24 h of incubation with Pd nanocrystals irradiated with the 808 nm NIR laser at 0.5 W cm^−2^ for 5 min per well; (3) only hydrogen therapy group, 24 h of incubation with PdH_0.2_ nanocrystals without the NIR irradiation; and (4) combined hydrogenothermal combination therapy group, 24 h of incubation with PdH_0.2_ nanocrystals irradiated with the 808 nm NIR laser at 0.5 W cm^−2^ for 5 min per well. After another 60 min of incubation in a CO_2_-free incubator, the cellular energy metabolism parameters were detected by the Extracellular Flux Analyzer (XF^e^ 24).

### Statistical analysis

All the data were presented as means ± s.d. To test the significance of the difference between sample groups, analysis by variance statistics was applied and a value of *P* < 0.05 was considered to be statistically significant.

## Electronic supplementary material

Supplementary Information

Reporting Summary

## Data Availability

The authors declare that the main data supporting the findings of this study are available within the article and its Supplementary Information. Extra data are available from the corresponding author upon request.
