# Supplementary material for: Local generation of hydrogen for enhanced photothermal therapy
Source: Nat Commun. 2018 Oct 12;9:4241. doi: 10.1038/s41467-018-06630-2 (PMC6185976; doi:10.1038/s41467-018-06630-2)
Supplement: Supplementary file 1 — Supplementary Information [file 41467_2018_6630_MOESM1_ESM.pdf]

Supplementary Information

Local generation of hydrogen for enhanced photothermal therapy

Penghe Zhao[1], Zhaokui Jin[1], Qian Chen[2,3,4], Tian Yang[1], Danyang Chen[1], Jin Meng[1], Xifeng Lu[1], Zhen Gu[2,3,4], Qianjun He[1]

[1] Guangdong Provincial Key Laboratory of Biomedical Measurements and Ultrasound Imaging, National-Regional Key Technology Engineering Laboratory for Medical Ultrasound, School of Biomedical Engineering, Health Science Center, Shenzhen University, No. 3688 Nanhai Road, Nanshan District, Shenzhen 518060, Guangdong, China

[2] Department of Bioengineering, University of California, Los Angeles, Los Angeles, CA 90095, USA

[3] California NanoSystems Institute, Jonsson Comprehensive Cancer Center, Center for Minimally Invasive Therapeutics,University of California, Los Angeles, Los Angeles, CA 90095, USA

[4] Joint Department of Biomedical Engineering, University of North Carolina at Chapel Hill and North Carolina State University, Raleigh, North Carolina, USA.

These authors contributed equally: Penghe Zhao and ZhaokuiJin.

Correspondence and requests for materials should be addressed to Q. H. (email: nanoflower@126.com) or Z. G. (email: guzhen@ucla.edu)

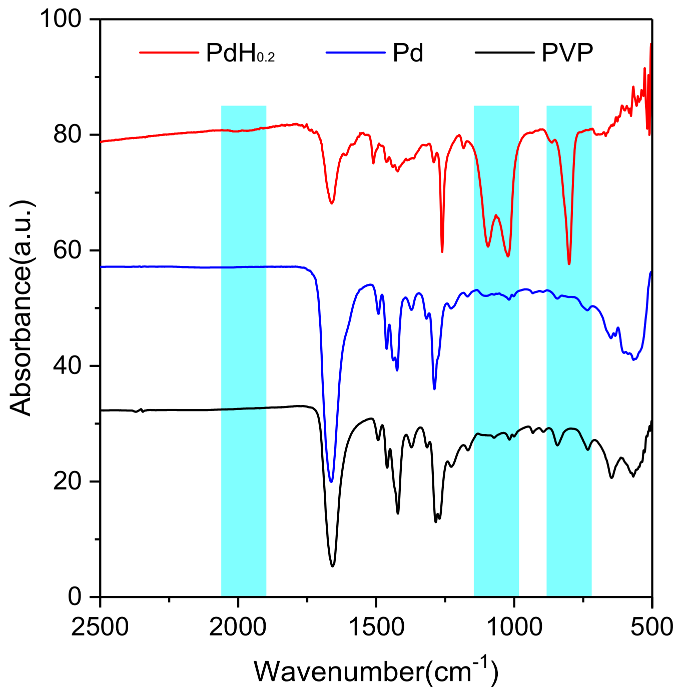

Supplementary Figure 1 | FT-IR spectra of PVP, PVP-coated Pd and $PdH_{0.2}$ nanocrystals.

Supplementary Discussion

Measurement of hydrogen release behavior of the $PdH_{0.2}$ nanocrystals by in-time UV monitoring. As to the UV monitoring, put 3 mL 0.04 mg mL^{-1} $PdH_{0.2}$ nanocrystal solution into the cuvette, irradiate the solution using an 808 nm NIR laser (1 W cm^{-2}), and immediately measure the UV spectra of the solution once every minute. From the change of the UV spectra (the band intensity below 410 nm increased firstly (Figure a) and then decreased (Figure b) with hydrogen release), we primarily decided that there could be a critical intermediate phase (PdHc).

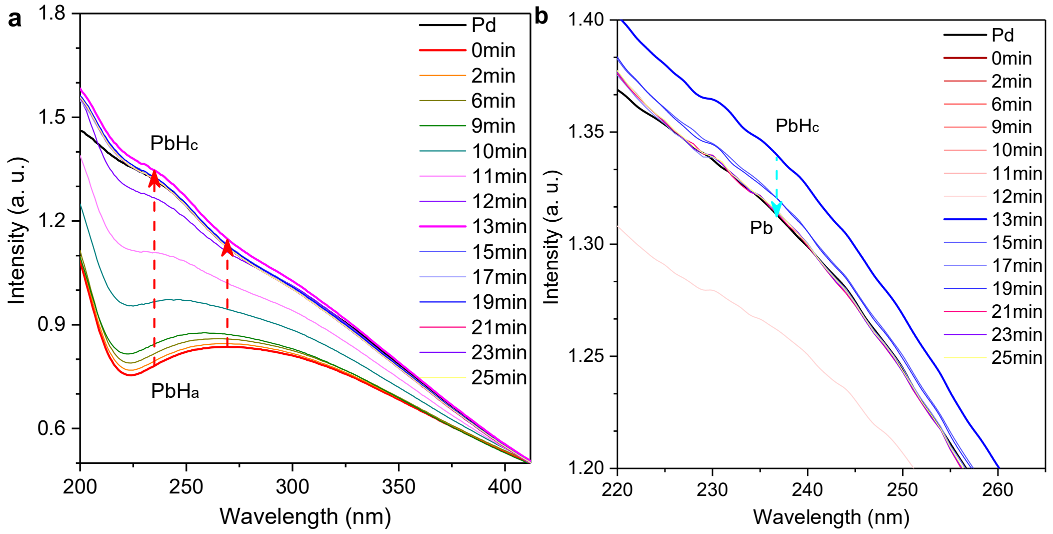

Supplementary Figure 2 | UV absorption spectra change of $PdH_{0.2}$ nanocrystals under the NIR irradiation.

Therefore, the decomposition process of $PdH_{0.2}$ was divided into two steps as following.

$$PdH_a \rightarrow PdH_c + \frac{a-b}{c} H_2 \uparrow \quad (1)$$

$$PdH_c \rightarrow Pd + \frac{c}{2}H_2 \uparrow \quad (2)$$

According to the Beer–Lambert law,

$$I = \sum_i^n \lambda \varepsilon_i C_i \quad (3)$$

For the first reaction, the increase in the intensity of 235 nm band with reaction is

$$\Delta I_1 = (\varepsilon_c - \varepsilon_a)\Delta C_1 \quad (4)$$

where ε_c and $\varepsilon_a$are the molar attenuation coefficient of PdHc and PdHa, respectively, ΔC_1 represents the decrease of PdHa and the increase of PdHc.

$$\therefore \frac{I - I_0}{C_{H1}} = \frac{I_{PdHc} - I_{PdHa}}{(a-b)/2} \quad (5)$$

where C_{H1}, I_{PdHa}, I_{PdHc} and I are the released hydrogen amount, the initial intensity of 235 nm band, the critical intensity of 235 nm band when PdHa decomposes completely into PdHc, and the measured intensity of 235 nm band, respectively.

The release percentage of hydrogen in the first reaction process is

$$Q_1 = \frac{C_{H_1}}{(a-b)/2} \quad (6)$$

$$\therefore Q_1 = \frac{C_{H_1}}{(a-b)/2} = \frac{I - I_{PdHa}}{I_{PdHc} - I_{PdHa}} \quad (7)$$

In the second reaction process, the release percentage of hydrogen is

$$\therefore Q_2 = \frac{C_{H_2}}{b/2} = \frac{I_{PdHc} - I}{I_{PdHc} - I_{Pd}} \quad (8)$$

where $C_{H2}$and I_{Pd} are the released hydrogen amount and the final intensity of 235 nm band when PdHc decomposes completely into Pd, respectively.

According to the in-time detected UV data (Fig. 2a), the release percentage of hydrogen can be calculated by using Equations (7) and (8), and plotted as a function of reaction time, as shown in Fig. 2b.

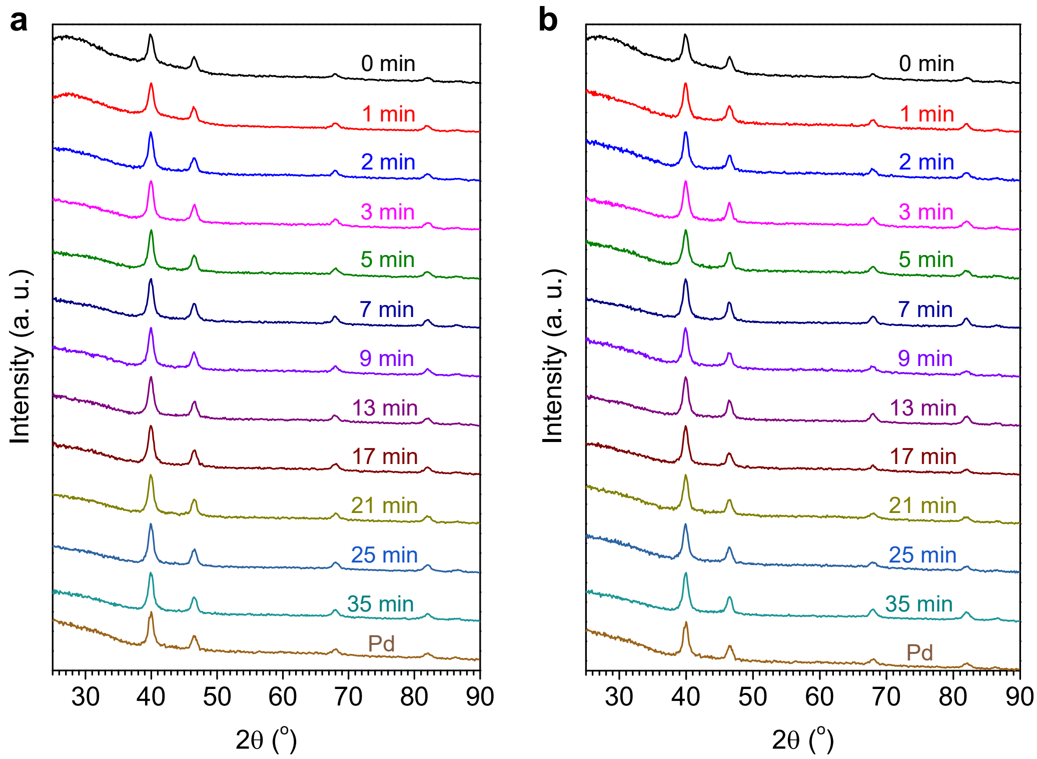

Supplementary Figure 3 | Real-time monitoring of hydrogen release from $PdH_{0.2}$ nanocrystals under the continuous irradiation of 808 nm NIR light at the power densities of 0.5 W cm^{-2} (**a**) and 0.2 W cm^{-2} (**b**), respectively.

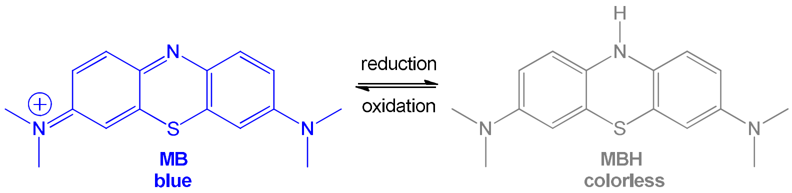

Supplementary Figure 4 | The hydrogen detection mechanism of the MB probe based on the color change during the redox reaction.

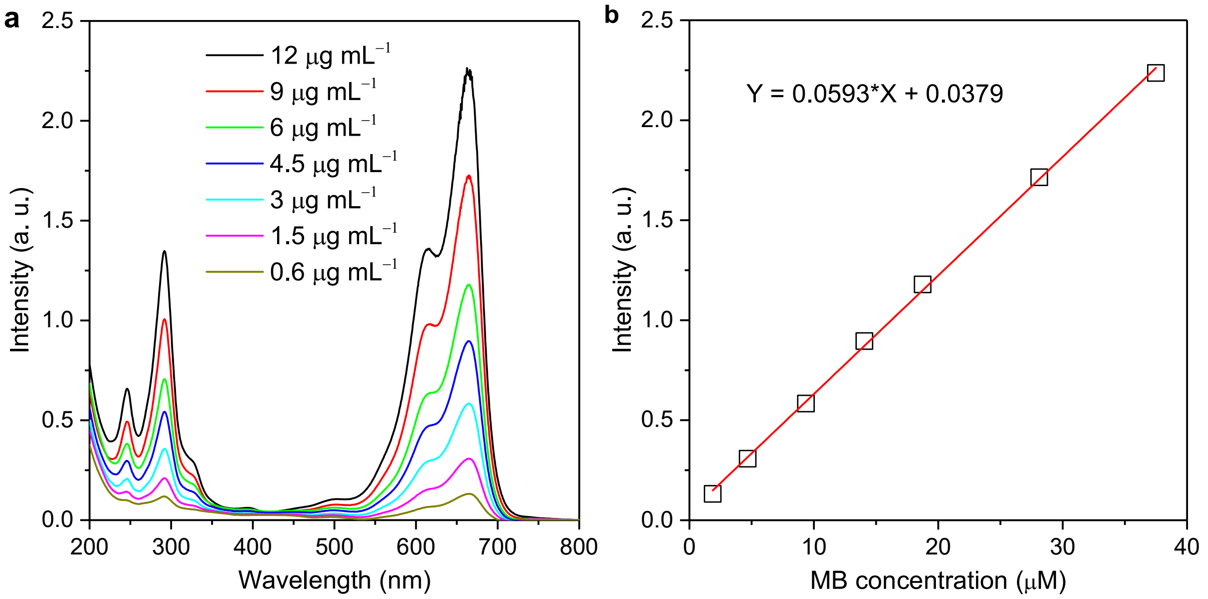

Supplementary Figure 5 | Absorption spectra of MB at different concentrations (a), and the linearly fitted standard curve of absorption intensity *vs* MB concentration (b).

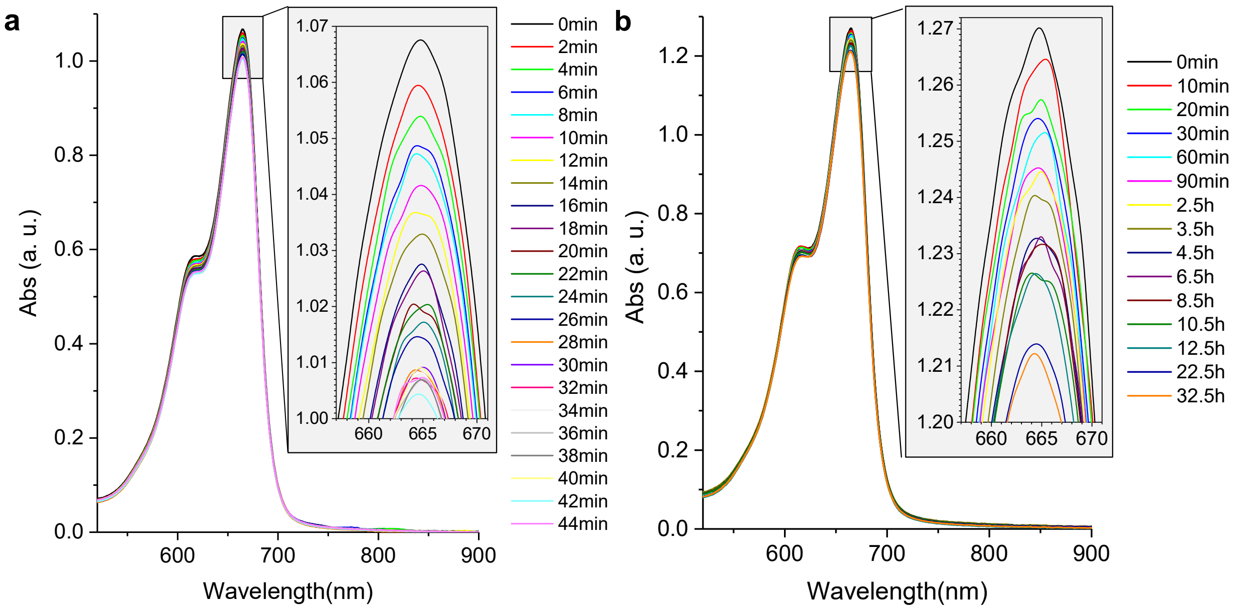

Supplementary Figure 6 | Time-dependent absorption spectra of the $PdH_{0.2}$+MB solution with NIR irradiation (a), and the $PdH_{0.2}$+MB solution without NIR irradiation (b).

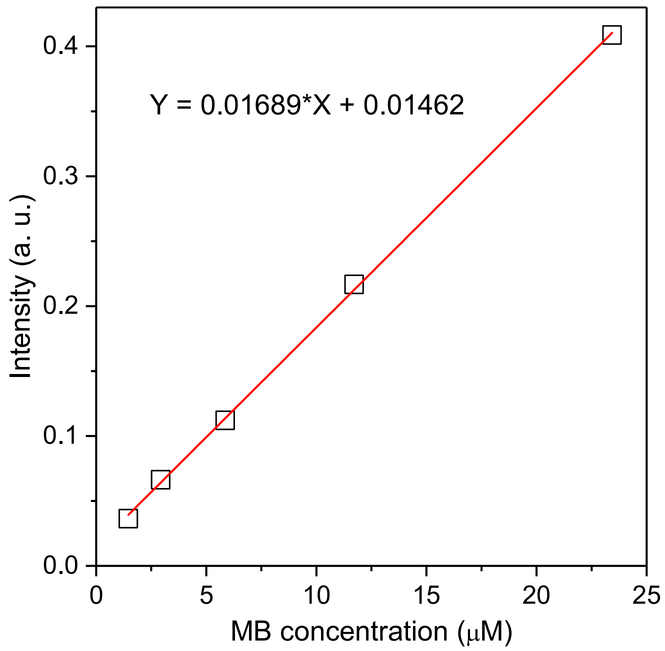

Supplementary Figure 7 | The linearly fitted standard curve of absorption intensity *vs* MB concentration, which was measured in 96-well plate using the microplate reader.

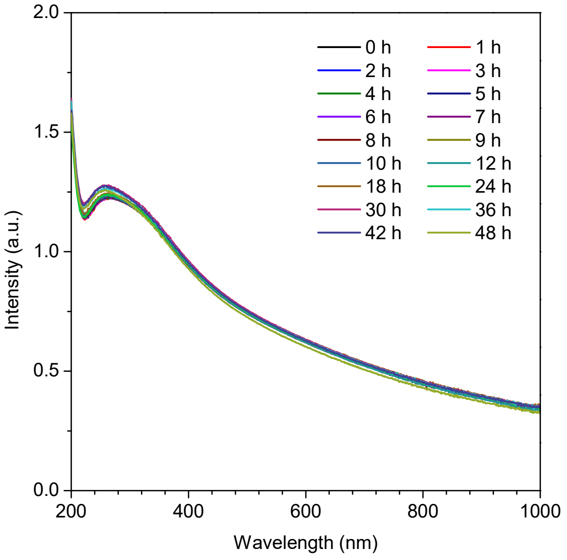

Supplementary Figure 8 | Stability evaluation of $PdH_{0.2}$ nanocrystals in the PBS by UV monitoring.

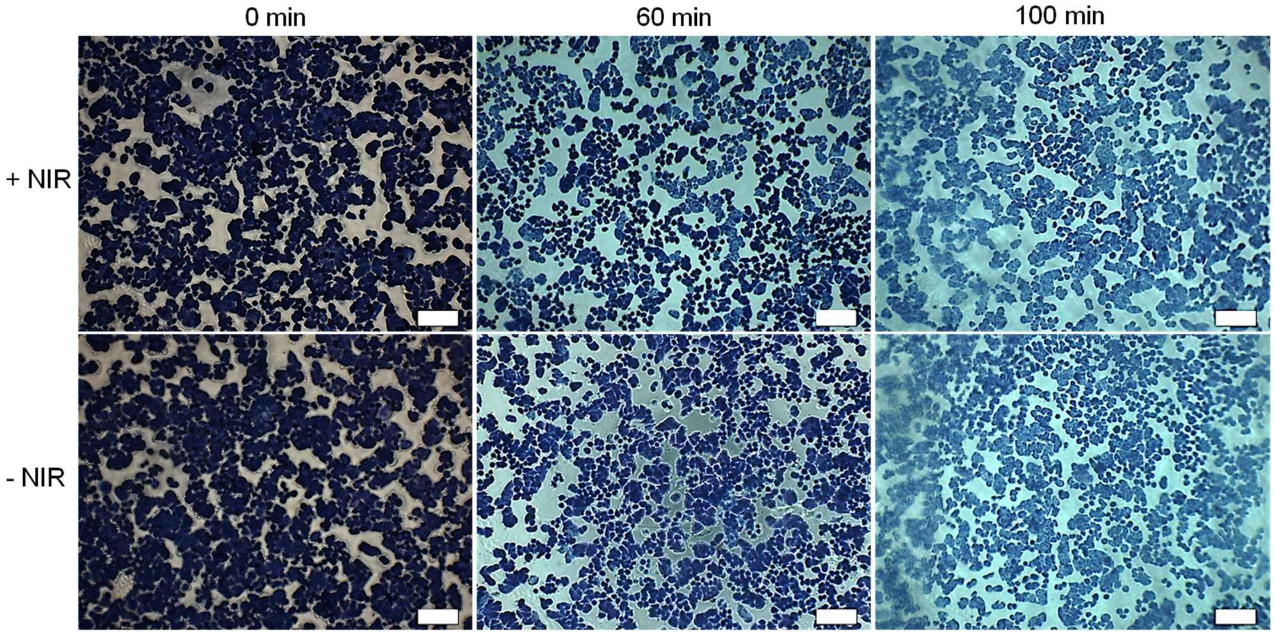

Supplementary Figure 9 | The NIR-accelerated decrease in blue color of MB-labeled cells with incubation time, reflecting the intracellular NIR-activated reducibility of $PdH_{0.2}$ nanocrystals. Scale bar, 100 μm.

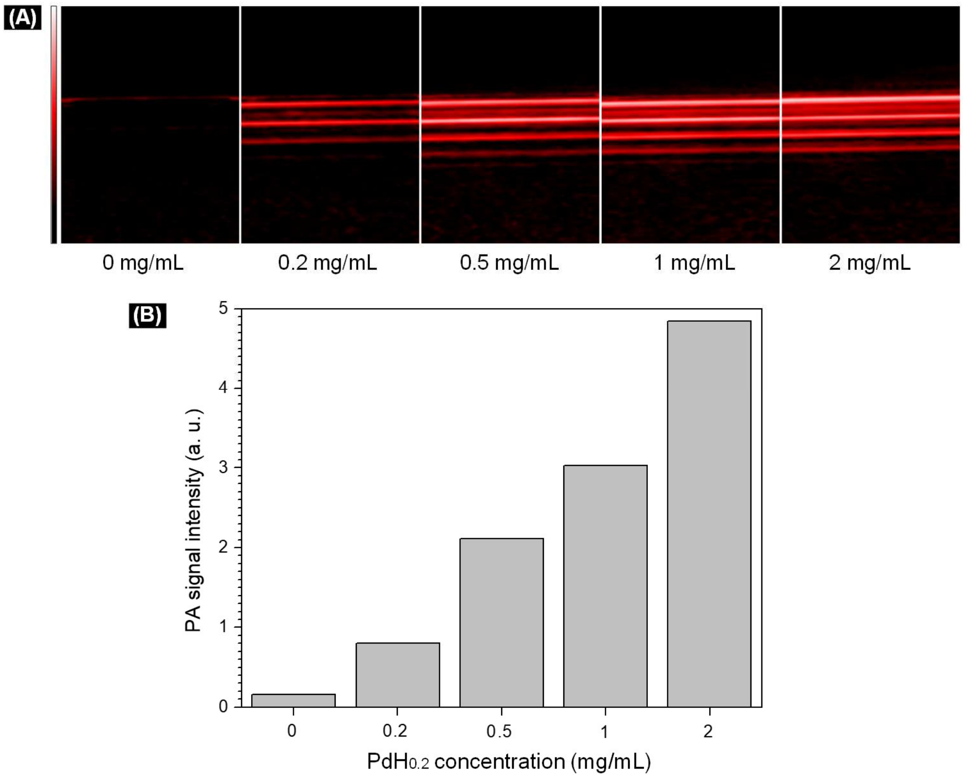

Supplementary Figure 10 | The dependence of PA imaging signal of $PdH_{0.2}$ nanoparticle solution on its concentration. Figure B is the quantitative statistical analysis of Figure A.

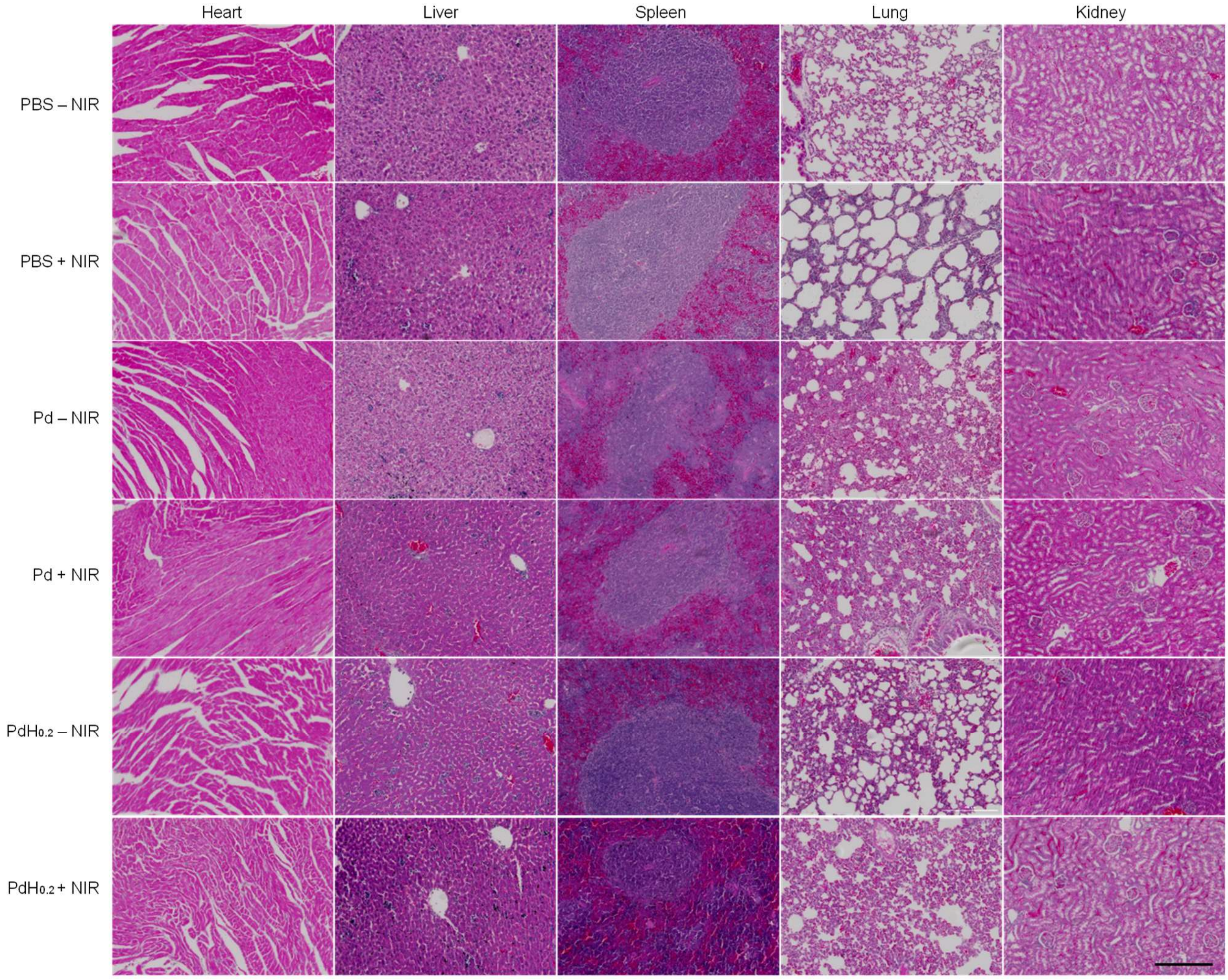

Supplementary Figure 11 | Histological examination of main organs (heart, liver, spleen, lung and kidney) from mice treated with the PBS–NIR blank control, the PBS+NIR control, the Pd–NIR group, the Pd+NIR group, the $PdH_{0.2}$–NIR group, and the $PdH_{0.2}$+NIR group, respectively, by the H&E staining method (Scale bars, 100 μm for all panels).

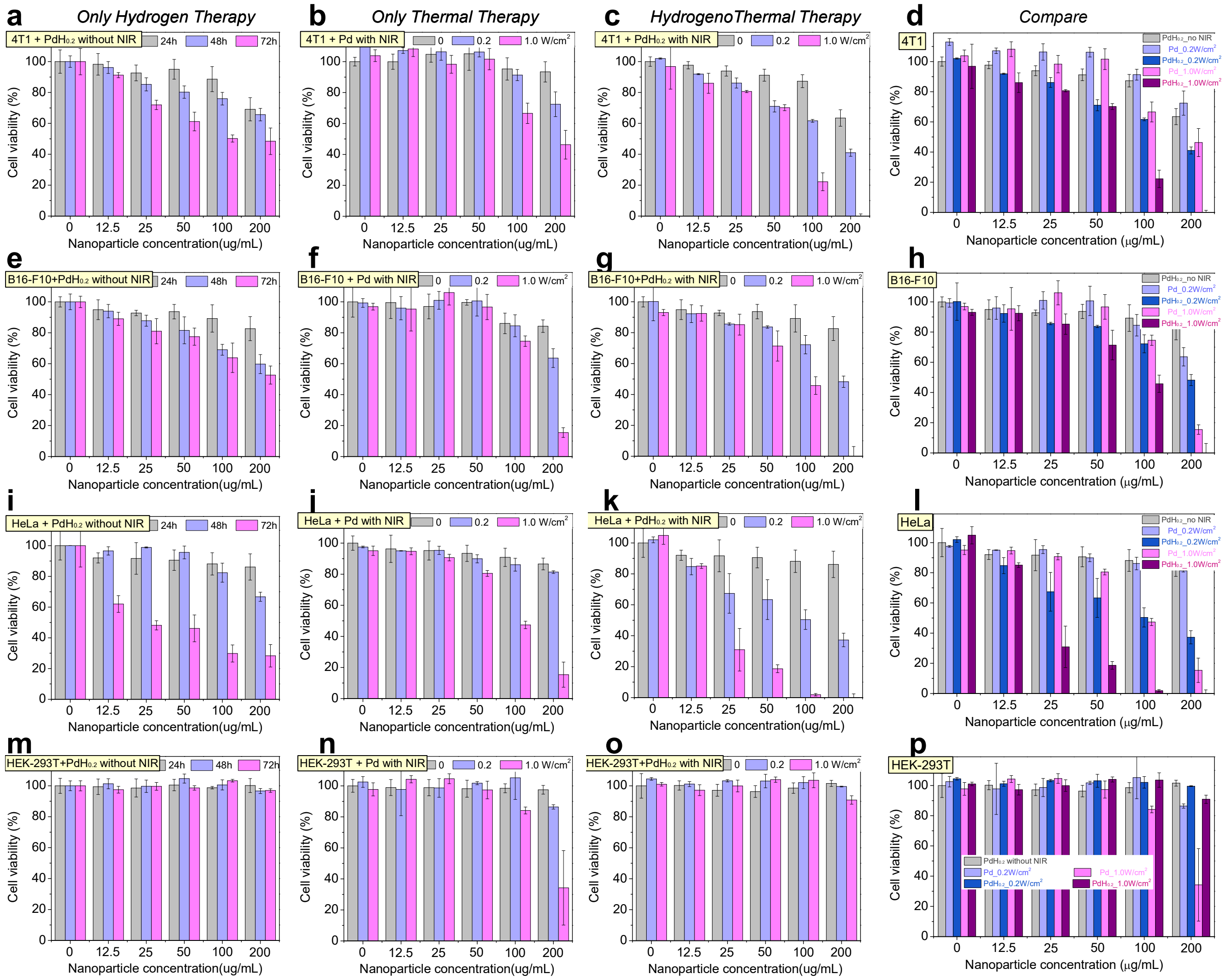

Supplementary Figure 12 | *In vitro* combined hydrogenothermal therapy efficacies of $PdH_{0.2}$ nanocrystals against various cancer cells. (**a**, **d**, **g**, **k**) Only hydrogen therapy by $PdH_{0.2}$ nanocrystals without NIR irradiation. (**b**, **e**, **h**, **m**) Only thermal therapy by simulating with Pd nanocrystals plus NIR irradiation. (**c**, **f**, **i**, **n**) Combined hydrogenothermal therapy by $PdH_{0.2}$ nanocrystals with NIR irradiation, against various cancer cells (4T1, B16-F10, HeLa cells) and HEK-293T normal cells (n = 8). Mean value and error bar are defined as mean and s.d., respectively.

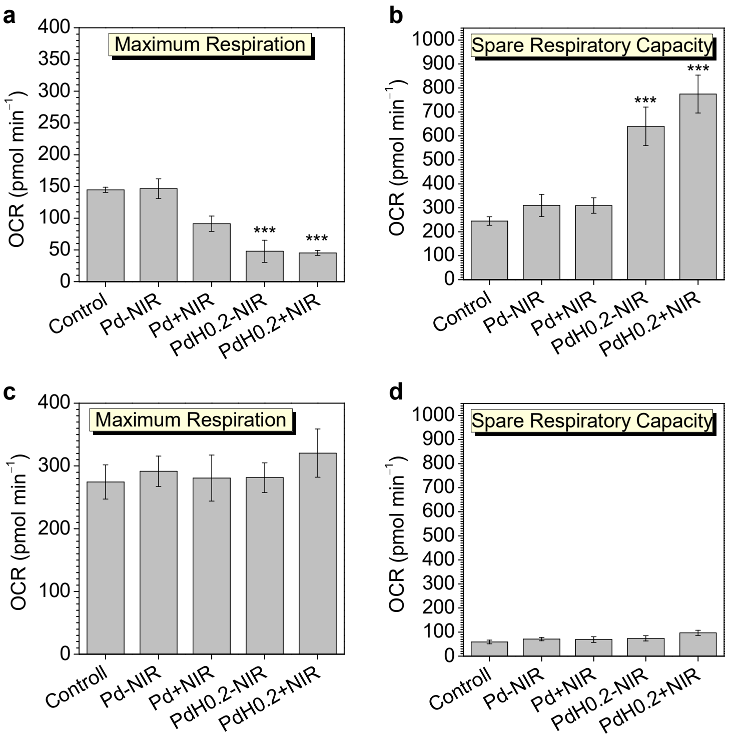

Supplementary Figure 13 | Maximum respiration and spare respiratory capacity of HeLa cells (a,b) and HEK-293T cells (c,d) (n = 4). P values were calculated by two-tailed Student's t-test (***P<0.005, **P<0.01, *P<0.05) by comparing other groups with the control group. Mean value and error bar are defined as mean and s.d., respectively.

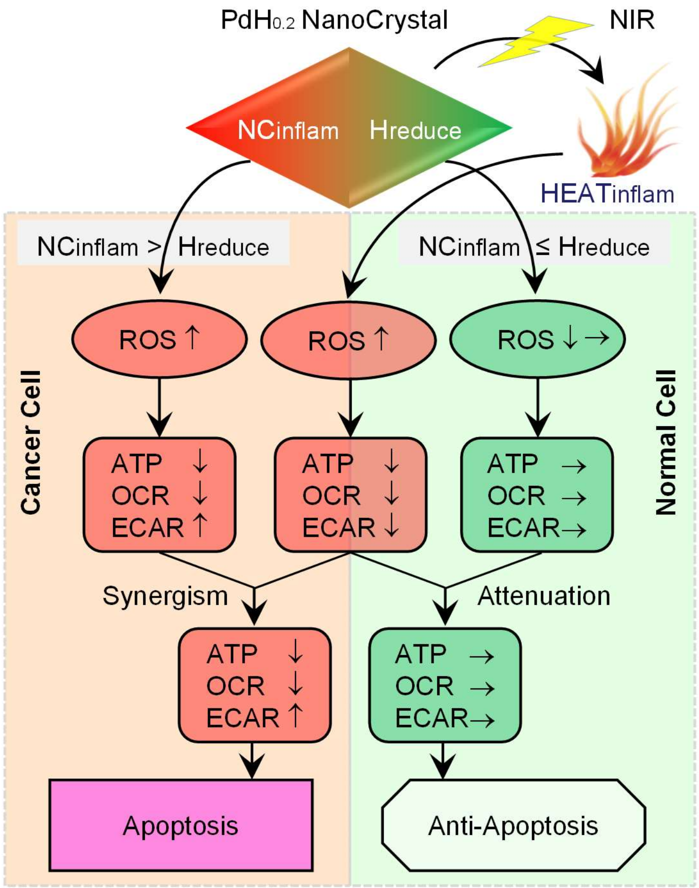

Supplementary Figure 14 | The synergism and attenuation mechanisms of hydrogenothermal therapy by $PdH_{0.2}$ nanocrystals under the NIR irradiation. NC_{infla}, H_{reduce} and $HEAT_{inflam}$ represented the inflammation response to $PdH_{0.2}$ nanocrystals, the counteraction of bio-reductive hydrogen against inflammation and the inflammation damage from heat, respectively.

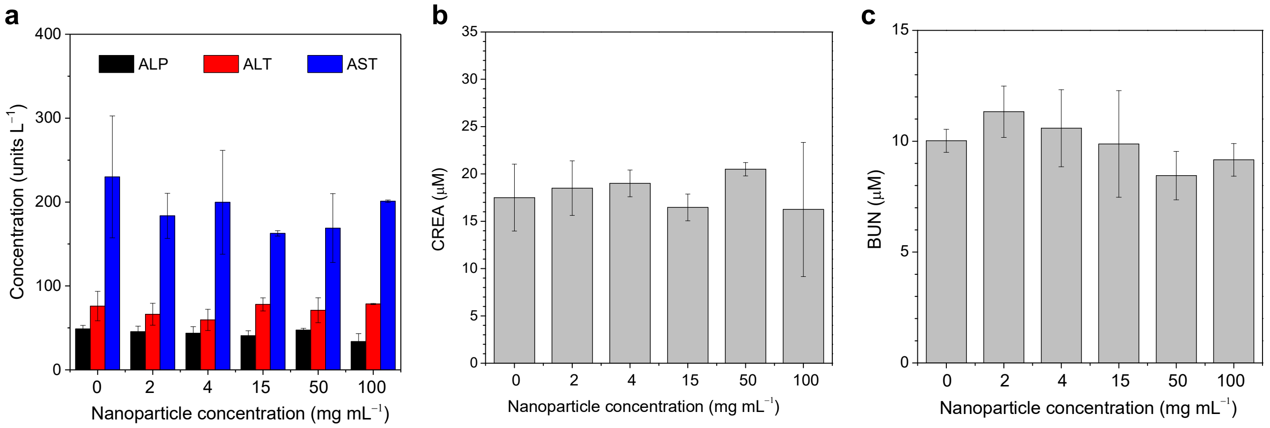

Supplementary Figure 15 | Blood biochemical analyses including liver functions (a) and kidney functions (b,c). ALP, alkaline phosphatase; ALT, alanine transaminase; AST, aspartate transaminase; CREA, creatinine; BUN, blood urea nitrogen (n =4). Mean value and error bar are defined as mean and s.d., respectively.

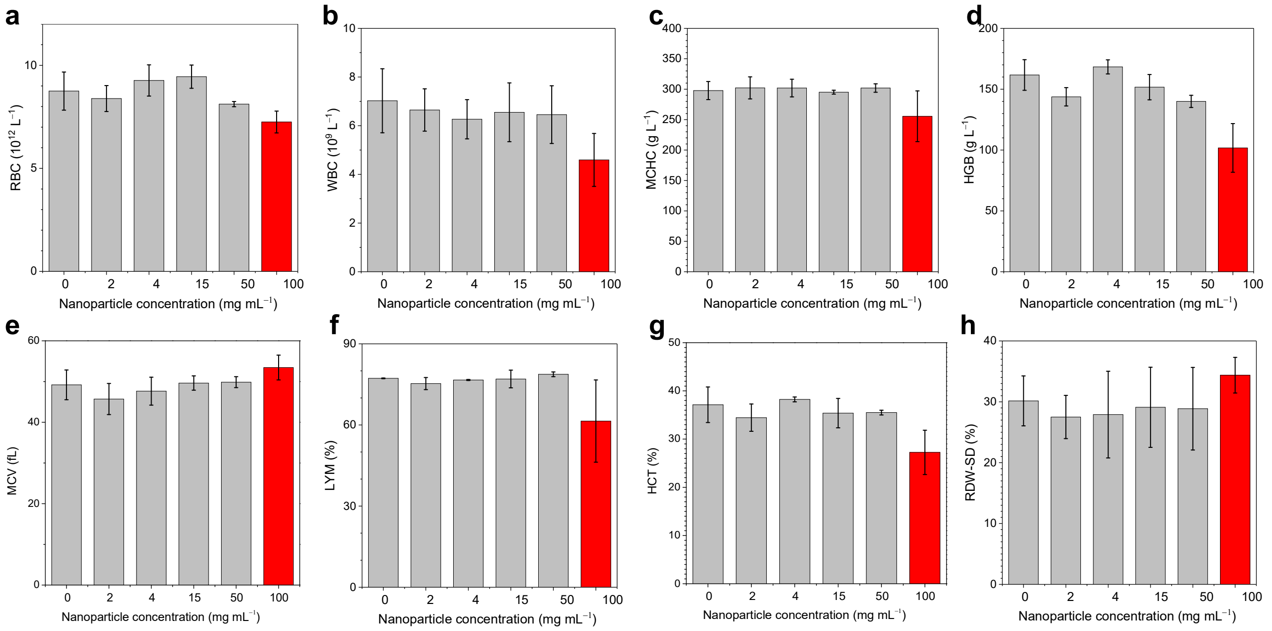

Supplementary Figure 16 | The evaluation of standard haematology markers including RBC (a), WBC (b), MCHC (c), HGB (d), MCV (e), LYM (f), HCT (g) and RDW-SD (h). RBC, red blood cells; HGB, haemoglobin; MCHC, means corpuscular haemoglobin concentration; LYM, lymphocytes percentage; MCV, means corpuscular volume; HCT, haematocrit; WBC, white blood cells; RDW-SD, red blood cell volume distribution width (n = 4). Mean value and error bar are defined as mean and s.d., respectively.

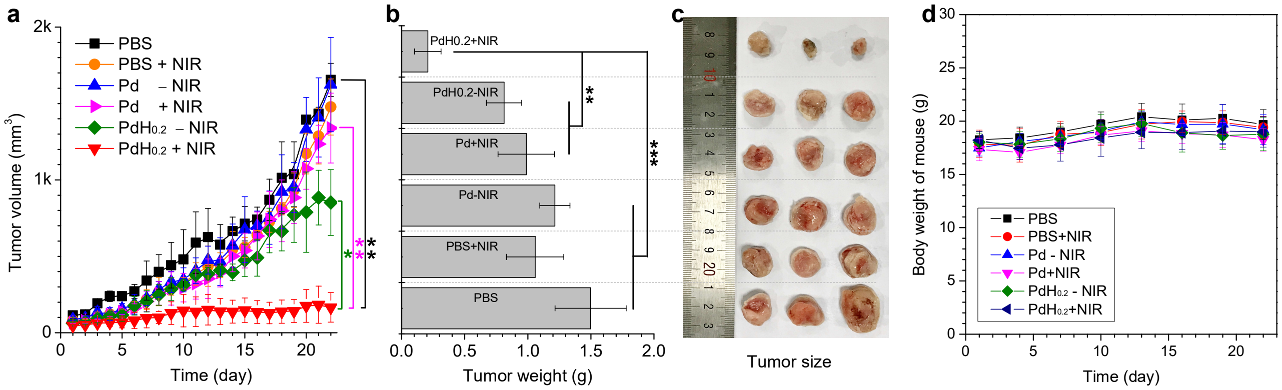

Supplementary Figure 17 | 4T1 tumour therapy efficacies of $PdH_{0.2}$ nanocrystals (1.0 W cm^{-2} NIR irradiation). (a) 4T1 tumour volume change during treatment. (b) 4T1 tumour weight comparison after 22 day treatment. (c) Digital images of corresponding 4T1 tumours. (d) The weight change of 4T1 tumour-bearing mice during treatment (n = 3). P values were calculated by two-tailed Student's t-test (***P<0.005, **P<0.01, *P<0.05) by comparing other groups with the $PdH_{0.2}$+NIR group. Mean value and error bar are defined as mean and s.d., respectively.

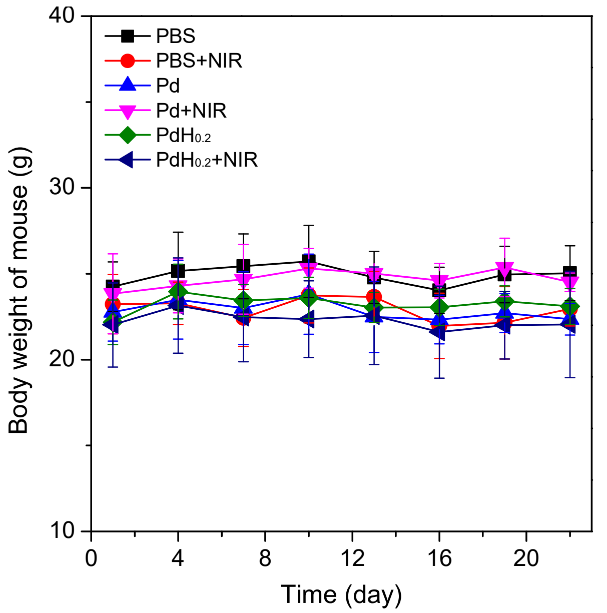

Supplementary Figure 18 | The weight change of 4T1 tumour-bearing mice during treatment (n = 6). Mean value and error bar are defined as mean and s.d., respectively.

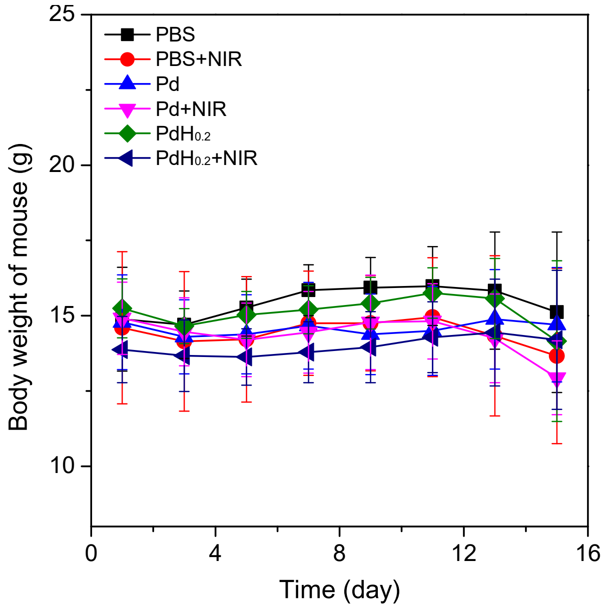

Supplementary Figure 19 | The weight change of B16-F10 tumour-bearing mice during treatment (n = 6). Mean value and error bar are defined as mean and s.d., respectively.
